# Incorporating inter-individual variability in experimental design improves the quality of results of animal experiments

**DOI:** 10.1371/journal.pone.0255521

**Published:** 2021-08-05

**Authors:** Marloes H. van der Goot, Marieke Kooij, Suzanne Stolte, Annemarie Baars, Saskia S. Arndt, Hein A. van Lith

**Affiliations:** 1 Faculty of Veterinary Medicine, Department Population Health Sciences, Section Animals in Science and Society, Utrecht University, Utrecht, the Netherlands; 2 Brain Center Rudolf Magnus, University Medical Center Utrecht, Utrecht, the Netherlands; Univerity of Texas at Austin, UNITED STATES

## Abstract

Inter-individual variability in quantitative traits is believed to potentially inflate the quality of results in animal experimentation. Yet, to our knowledge this effect has not been empirically tested. Here we test whether inter-individual variability in emotional response within mouse inbred strains affects the outcome of a pharmacological experiment. Three mouse inbred strains (BALB/c, C57BL/6 and 129S2) were behaviorally characterized through repeated exposure to a mild aversive stimulus (modified Hole Board, five consecutive trials). A multivariate clustering procedure yielded two multidimensional response types which were displayed by individuals of all three strains. We show that systematic incorporation of these individual response types in the design of a pharmacological experiment produces different results from an experimental pool in which this variation was not accounted for. To our knowledge, this is the first study that empirically confirms that inter-individual variability affects the interpretation of behavioral phenotypes and may obscure experimental results in a pharmacological experiment.

## 1. Introduction

In preclinical experimental animal research, inter-individual variability in phenotypic response is a major source of within-group variability that may negatively affect the power of animal experiments and the reproducibility of their outcomes [1–3]. The exact constitution of inter-individual variability (also referred to as the third component [2] or phenotypic variation [3]) is poorly understood. It is generally accepted however, that the expression of inter-individual variability is the net result of complex interactions between genetic and environmental factors that are partly modulated by epigenetic processes [3,4]. As a result, quantitative traits have even been shown to vary between individuals of the same mouse inbred strain, despite extensive environmental standardization and the use of genetically and microbiologically defined mice of similar age and sex [1,3].

Inter-individual variability however is often not actively accounted for in the design of animal experiments [3]. Traditionally, this type of variation was regarded as part of a larger source of unwanted noise, falling within the same category as other sources of extraneous noise (i.e. measurement error) and unanticipated environmental effects [3]. In contrast to these other sources however, inter-individual variability has been shown to be relatively robust to standardization efforts, distinguishing it from mere noise [2]. Therefore, an increasing body of research focuses on the identification of methods that consider inter-individual differences by systematically incorporating this variation in experimental design and statistical analysis [3,5–12].

The importance of incorporating inter-individual variability in the design of animal experiments has become especially acknowledged in animal models of behavioral dysfunction (e.g., anxiety, depression [5,13,14], post-traumatic stress disorder, [15,16] but also addiction [17] etc.). In humans, the susceptibility between individuals to develop a particular disorder, as well as the response to treatment is known to vary substantially between individuals [13]. Considering this variability may therefore not only improve reproducibility between studies, it may also contribute to an improved understanding of the mechanisms that underlie such inter-individual variability in human patients [5,13]. In this field, several strategies exist to incorporate inter-individual variability as a variable [17,18]. The most prominent strategy considers inter-individual variability between subpopulations within an experimental pool (mostly outbred stocks of rats and mice) by separating experimental animals whose expression of a particular trait (i.e. anxiety, activity) lies on opposing ends of a phenotypic distribution, for example by means of a median, tertiary, quartile split [5,17,18]. Interestingly, this use of selection strategies has indirectly demonstrated how existing subpopulations within an experimental pool may mask the detection of overall group effects, thereby providing examples of how inter-individual variability may confound experimental outcomes [14]. Barbelivien et al. [19], for example identified five sub-populations of Long Evans outbred rats that were characterized by differential levels of baseline impulsive choice behavior. Subsequent administration of d-amphetamine only affected impulse choice behavior when these baseline differences were accounted for, while no effect was found when all animals were pooled in the analysis.

A fundamental principle of good design of animal experiments is that all variables should be controlled except that due to treatment and that all treatment and control groups should be identical, with minimal within-group variability [20,21]. Following these principles, accounting for inter-individual variability in the composition of experimental groups should result in better matched individuals regarding control and treatment, thereby improving the experimental design and the quality of the results. To our knowledge however, the extent to which active incorporation of inter-individual differences in the composition of experimental groups affects the outcome of preclinical animal experiments, has never been directly tested.

In this study, we therefore compared the outcomes of an experimental design in which this inter-individual variability was accounted for, to a design in which this variability was not accounted for, to empirically assess to what extent active incorporation of inter-individual variability indeed alters the interpretation of a standard pharmacological experiment when evaluating the effects of an anxiolytic compound on anxiety-related behavior. To do so we defined the behavioral phenotype of experimental animals on an individual level in a pre-experimental period, and subsequently incorporated this information in the design and statistical analysis of our study.

A priori identification of subgroups within an experimental pool is also common in the aforementioned selection strategies. A disadvantage of these selection strategies however is that in the majority of these studies inter-individual variability is established by means of an artificially predetermined quantile (but see [22,23] for exceptions). Median split strategies may lead to a loss in resolution and power as every value above and below the mean is considered equal, regardless of its position on the phenotypic distribution [24]. Furthermore, strategies considering upper and lower quantiles only include the outer ends of a phenotypic distribution, rather than the entire study population [25]. These criteria contrast with the generally accepted conceptualization of human psychopathology as a continuum [26], which warrants the exploration of subgroups across the entire study population on the basis of variability in the data itself, rather than a predefined criterion [14].

In the present study we therefore used a data-driven clustering approach to identify meaningful subpopulations within our experimental pool (see below). Furthermore, instead of outbred stocks, we assessed inter-individual variability in mouse inbred strains. As outlined above, inter-individual differences in spontaneous behavior have been repeatedly demonstrated within mouse inbred strains, and have been found to be consistent over time within individuals [27–29]. In fact, inbred strains of mice have been demonstrated to be just as variable as outbred stocks of this species [30].

We expanded on a series of previous studies in which we found that our phenotype of interest, behavioral habituation of anxiety-related responses, may differ within BALB/c, C57BL/6 and various 129 substrains [31,32]. These studies measured behavioral habituation as the change in anxiety and activity related behavior after repeated exposure to a mild aversive stimulus (the modified Hole Board (mHB)). Anxiety is typically regarded as a complex behavioral construct that is expressed by both anxiety-related and activity behaviors [33–35]. Multivariate cluster analyses on the resulting individual response trajectories identified two clusters in which mice grouped together across both anxiety and activity related dimensions: individual response types. These response types were of differential adaptive value, and were displayed by individuals of all three strains.

In the present study, we used the same experimental assay and statistical procedure to first behaviorally characterize BALB/c, C57BL/6 and 129S2 mice on their individual response type (pre-experimental period). Next, we designed a pharmacological experiment in which we systematically incorporated this factor in the composition of our experimental groups. We used a complete randomized block design with four replicates (‘mini-experiments or blocks’, [36,37]) and systematically incorporated experimenter, besides inbred strain, as a heterogenization factor to improve the generalizability of our results, as suggested by Richter [38].

Previous research showed that anxiolytic compounds may improve behavioral habituation of anxiety responses in the mHB [39]. In this experiment we evaluated the effectiveness of dexmedetomidine as an anxiolytic. In humans this highly selective alpha 2A-adrenergic receptor agonist is reported to exert anxiolytic effects when administered as an analgesic sedative [40]. In mice this compound is used in search for brain mechanisms behind anxiety related behavior, because the alpha 2A-adrenoceptor system is known to play a crucial role in acute neuropsychological stress responses [41].

## 2. Materials and methods

### 2.1. Ethical statement

The experimental protocol was approved by the Central Animal Experiments Committee (CCD), the Hague, the Netherlands (CCD approval numbers: AVD1080020172264 and AVD1080020172264-1). The resolution for approval was reached on the basis of the Dutch implementation of EU directive 2010/63/EU (Directive on the Protection of Animals Used for Scientific Purposes). The experiment was conducted according to the Dutch ‘Code on Laboratory Animal Care and Welfare’. Furthermore, the present animal study is reported to the best of our abilities according to the revised ARRIVE guidelines (ARRIVE 2.0; https://www.nc3rs.org.uk/revision-arrive-guidelines [42,43]).

### 2.2. Animals and housing

This study tested naïve males of three mouse inbred strains: BALB/cAnNCrl (hereafter C, *n* = 59, white (albino), strain code = 028), C57BL/6NCrl (B6N, *n* = 60, black, strain code = 027) and 129S2/SvPasCrl (129S2, *n* = 60, agouti, strain code = 287). One additional C mouse died due to reasons unrelated to the study and was not tested.

Furthermore, an additional number of 15 naïve males (*n* = 5/strain) were used to establish the required dose of pharmacological treatment in a pilot study. One B6N mouse died due to reasons unrelated to the pilot study and was not tested. The total number of animals used in the present study amounted to 179 + 14 = 193. The sample size was determined using the software by Lenth (www.stat.uiowa.edu/~rlenth/Power). To eliminate possible effects of estrous cycle on behavioral variables [44], only male mice were used in this study.

Animals were bred by and purchased from Charles River Germany (Sulzfeld, Germany). All mice were 7 weeks old upon arrival (body weight (g), mean ± standard error of the mean (SEM) and range: C, 20.4 ± 0.20 and 13.5–23.4; B6N, 21.0 ± 0.20 and 17.5–24.0; 129S2, 24.1 ± 0.27 and 19.3–28.3). Animals were housed at the Central Laboratory Animal Research Facility of Utrecht University. Testing took place in the same rooms as where the animals were housed, and test equipment was placed in each room prior to arrival of the animals.

Mice were housed individually to reduce aggression and to avoid a potential confounding effect of aggression in (part of) the study population [45,46]. Mice were housed in Macrolon Type II L cages (size: 365 x 207 x 140 mm, floor area 530 cm^2^, Techniplast, Milan, Italy) with standard bedding material (autoclaved Aspen Chips, Abedd-Dominik Mayr KEG, Köflach, Austria) and a tissue (KLEENEX^®^ Facial Tissue, Kimberley-Clark Professional BV, Ede, the Netherlands) and a plastic PVC shelter as enrichment (Plexx BV, Elst, the Netherlands). Food (CRM, Expanded, Special Diets Services Witham, UK) and tap water were available *ad libitum*. Upon arrival mice were randomly allocated to one of two laboratory animal housing rooms for a habituation period of 17 days under a reversed 12 h light/12 h dark cycle (lights off at 7:00 AM) with a radio playing constantly as background noise. The number of mice per strain was similar between the two testing rooms. Relative humidity (mean percentage ± SEM) was controlled (room A: 53.5% ± 2.44; room B: 54.8% ± 2.56) with a ventilation rate of 15–20 changes/hour (both rooms) an average room temperature (mean°C ± SEM) of 21.7°C ± 0.23 and 21.9°C ± 0.40 for room A and B, respectively. The mice were handled three times a week during the habituation period by the same two experimenters that conducted the behavioral observations. Handling mice included picking up the mouse at the base of the tail and placed briefly on top of the home cage or on the arm of the experimenter to accustom them to test procedures.

### 2.3. Modified Hole Board

Mice were tested in the modified Hole Board (mHB), a test for assessment of unconditioned behavior that combines characteristics of an open field, a hole board and a light-dark box [47]. This assay is designed for analyzing a range of anxiety and activity related behaviors and as such is suitable for a complete phenotyping of complex behavioral constructs, such as behavioral habituation of anxiety responses. The mHB has been described extensively elsewhere [48] and is only briefly explained here.

**Fig 1:**
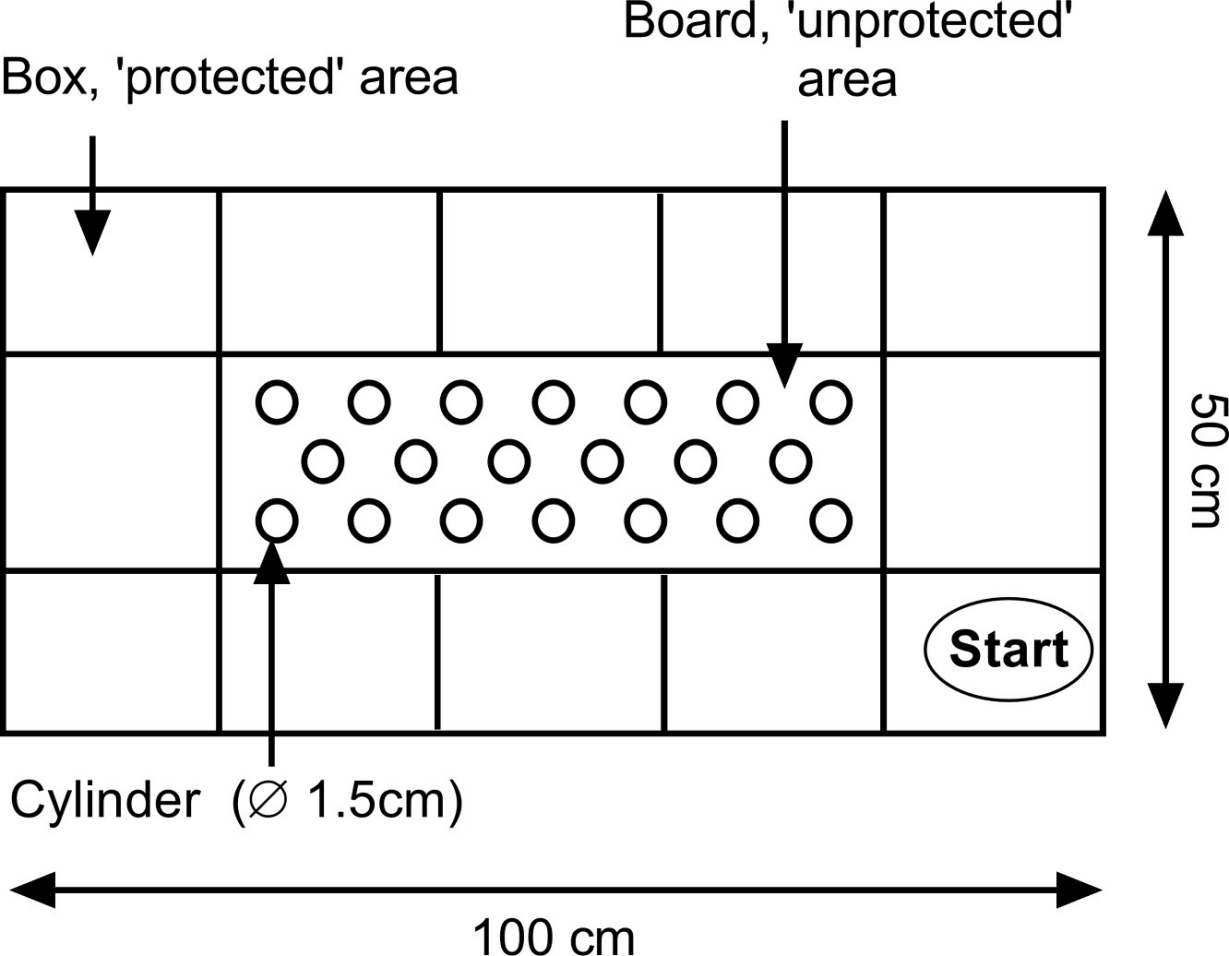
Schematic overview of the modified Hole Board.

Fig 1 presents a schematic overview of the mHB. The apparatus consists of a grey PVC opaque box (100 x 50 x 50 cm) with a board made of the same material (60 x 20 x 20 cm) functioning as an unprotected area, as it is positioned in the center of box. The board stacks 20 cylinders (diameter 15 mm) in three lines. The area around the board is divided into 10 rectangles (20 x 15 cm) and 2 squares (20 x 20 cm). The periphery was illuminated with red light (1–5 lux) and functioned as the protected area. In contrast, the central board was illuminated by an additional stage light in order to increase the aversive nature of the central (unprotected) area. Light intensity (mean lux) was 147 and 151 lux in room A and B, respectively.

### 2.4. Experimental protocol

Experimental Phase 1 was used to characterize the mice on their individual response type. In Experimental Phase 2, we designed a pharmacological experiment in which we systematically incorporated this factor (individual response type) in the composition of our experimental groups.

#### Experimental Phase 1

In order to characterize all 179 mice on their individual response type, the behavior of each individual was repeatedly assessed in the mHB. Each mouse was tested individually for a total of five subsequent trials. Each trial lasted 5 minutes. Behavioral testing occurred between 10 AM and 2 PM, during the active phase of the animals. Data was collected during 6 weeks, with a total number of *n* = 30 (*n* = 10/strain) animals per week. Test order was randomized across strains within each week.

At the start of the first trial, mice were transferred from the home cage to the mHB and always placed in the same corner, facing the central board. During the test, mice were allowed to freely explore the mHB-set up. Between trials mice were picked up by the tail and transported back to their home cage. The mHB was subsequently carefully cleaned with water and a damp towel before the next trial commenced. Behavior was scored live using the software Observer version 12.5 (Noldus Technology, Wageningen, the Netherlands). In addition, trials were recorded on video camera for raw data storage. Behavioral observations were conducted by two trained observers, each of which always tested in the same housing room. For obvious reasons, it was not possible to perform the observations blinded with respect to strain (due to the coat color of the animals, see section 1.2. Animals and Housing). Inter-observer reliability was established at a strong level [49] with an average Cohen’s *κ* = 0.80 (range 0.71–0.95) over a percentage agreement of 84.27% (range 76.68–96.35) for frequency scores. For duration scores the inter-observer reliability was established at a strong level, with an average Cohen’s *κ* = 0.88 (range 0.79–0.95) over a percentage agreement of 92.45% (range 86.20–97.28).

In addition to behavioral observations, circulating corticosterone levels (pCORT) were assessed for each individual mouse at three different time points: one week prior to behavioral testing, directly after the last mHB trial and one week after behavioral testing. These samples were collected with the intention to include pCORT trajectories in our cluster analysis used for classification of the individual response types. However, due to procedural errors during blood sampling and laboratory assay of the plasma there were a substantial number of missing or excluded samples (*n* = 30 samples, of *n* = 28 individuals). To maintain a sufficient sample size and power for the second part of our experiment, individual characterization of mice was therefore only based on their behavioral response.

#### Experimental Phase 2

In Experimental Phase 2, we systematically incorporated the factor individual response type in the composition of our experimental groups in a pharmacological experiment. A total number of 96 mice were matched to pairs (*n* = 48 pairs, *n* = 16 pairs/strain). The factor individual response type was systematically included by matching half of these pairs on body weight and their individual response type (*n* = 24 pairs, *n* = 8 pairs/strain). This balanced pool represented an experimental design in which individual response type was taken into account.

The remaining half of the pairs were matched on body weight only (*n* = 24 pairs, *n* = 8 pairs/strain). This unbalanced pool mimicked a regular experimental setup in which individual response type was not controlled for. In theory, not accounting for individual response type may result in pairs that share the same response type, or pairs that differ in individual response type. The matched pairs in the unbalanced pool therefore consisted of pairs that shared the same individual response type (62.5%, *n* = 13), and pairs that did not (37.5%, *n* = 9).

Within each pair, one mouse was treated with an anxiolytic, while the other served as control. Treatment and control were assigned randomly within pairs. Treatment consisted of an intra-peritoneal injection (*i*.*p*.) with dexmedetomidine (Dexdomitor®, 10 μg/kg, 100 μl; Orion Corporation-Orion Pharma, Espoo, Finland). Often used as a sedative-analgesic agent, this pharmacon is a highly selective α_2_-adrenergic receptor antagonist that also poses anxiolytic properties [50]. The selected dose was based on a pilot study in which this dose produced behavioral changes but no sedative effect. Control mice of each pair received a saline injection (NaCl, 0.9%, 100 μl, *i*.*p*). Treatment and control were assigned randomly within pairs.

**Fig 2:**
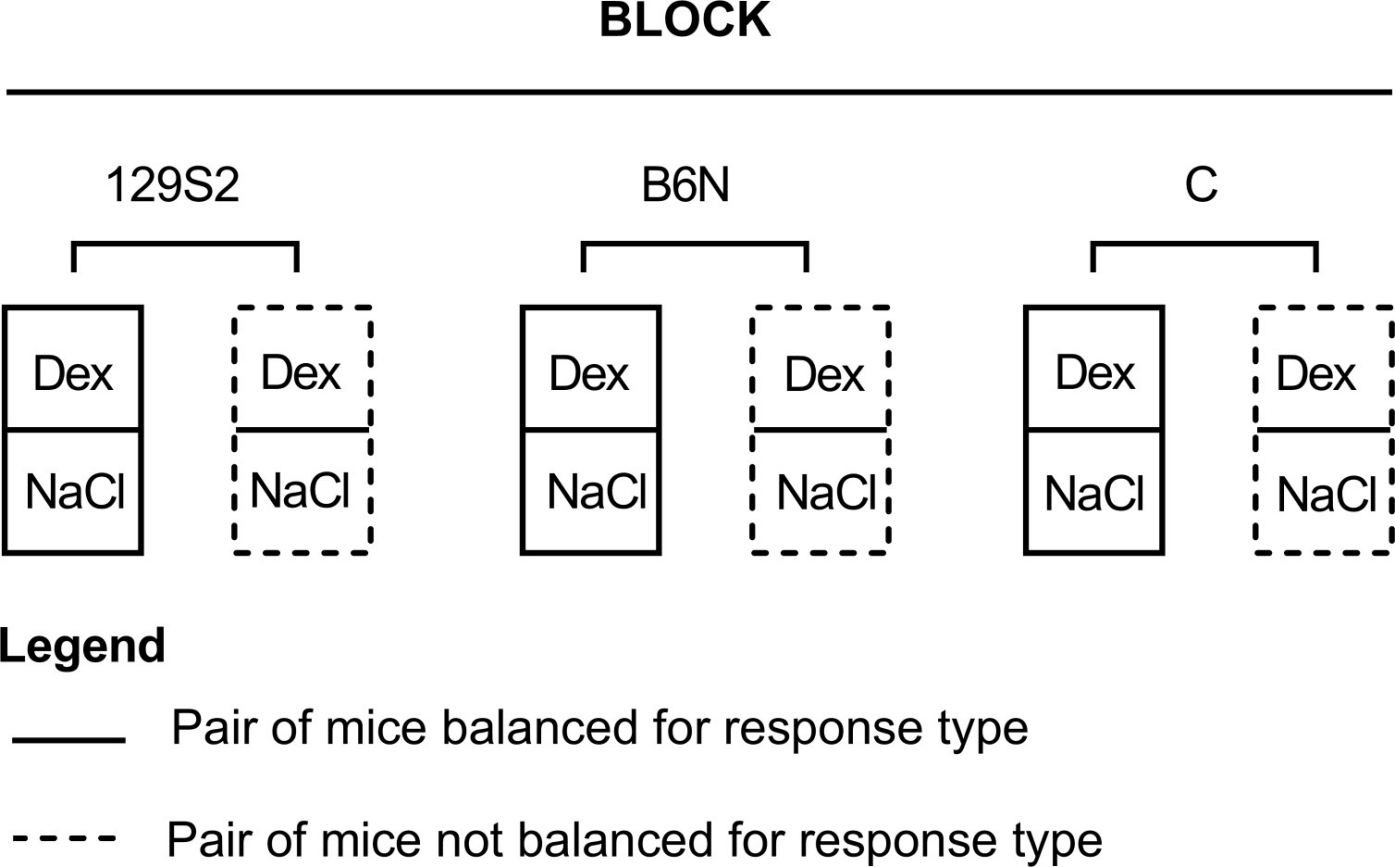
Schematic overview of the 2 (treatment) x 3 (strain) x 2 (experimenter) factorial complete randomized block design used in the pharmacological experiment. Overview represents a single block, for one experimenter. Pairs in the balanced condition were matched on weight and individual response type. Pairs in the unbalanced condition were only matched on weight. The unbalanced condition mimicked a ‘regular’ animal experiment in which individual response type is not taken into account in assignment to experimental groups. Pairs in the unbalanced condition could therefore consist of one animal from cluster A, and one animal from cluster B, or consist of two animals from the same cluster. Pairs in the balanced condition only consisted of two animals of the same cluster. Within each of 4 blocks (4 testing days) for each experimenter, all pairs were matched within strain, and within experimenter. Dex = treatment with dexmedetomidine (Dexdomitor®, 10 μg/kg, 100 μl, *i*.*p*); NaCl = control treatment with saline (NaCl, 0.9%, 100 μl, *i*.*p*).

Pairs of mice were tested over a period of 4 consecutive days, with a weekend in between (thu-fri-mo-tue). The experiment was designed as a complete randomized block design in which each test day was treated as a separate block. The numbers of pairs were maintained equal between test days, between strains and between experimenter. This amounted to 1 balanced, and 1 unbalanced pair (2) per strain (3), per experimenter (2) per block (4), resulting in 48 pairs. Fig 2 presents a schematic representation of the distribution of pairs within a single block (test day), for one experimenter.

At the start of each test day all animals of that test day were weighed to determine the injection volume, 60 minutes prior to start of the first mHB trial. Each mouse was tested individually for a single mHB trial, which lasted for 5 minutes. The experimental procedure of mHB testing was the same as in Experimental Phase 1. Behavioral testing in the mHB again occurred between 10 AM and 2PM. All mice received an intra-peritoneal (*i*.*p*.) injection (dexmedetomidine or saline) 30 minutes before being placed in the box compartment of the mHB. All injections were given by an experienced technician, who was not involved in the behavioral observations. Pairs of mice were tested after one another, and testing of pairs of the balanced and the unbalanced pool was alternated. Test order of pairs was randomized across strain, experimenter and test day. Behavioral observations were conducted by the same two experimenters, each in the same housing room, as in Experimental Phase 1, using the same ethogram and the same software. These experimenters were blind to treatment, pair and whether the pair was balanced or unbalanced on individual response type.

### 2.5. Behavioral variables

#### Experimental Phase 1 and 2

Behavioral patterns of mice were assessed by scoring behaviors listed in Table 1. From these observations, several parameters for avoidance behavior, exploration and locomotion were computed (Table 1).

**Table 1:** Behavioral parameters measured in the modified Hole Board.

Behavioral dimension	Behavioral parameter	Description of mouse behavior
Avoidance behavior	Total number of board entries	Mouse on the central board
	Latency until the first board entry	
	Percentage of total time spent on board	
Exploration	Total number of rearings in the box	Rearing on hind paws in the box
	Latency until the first rearing in the box	
	Total number of rearings on the board	Rearing on hind paws on the board
	Latency until the first rearing on the board	
	Total number of hole explorations	Exploration of a cylinder (hole) on the board
	Latency until the first hole exploration	
	Total number of hole visits	Nose-poking into a cylinder (hole) on the board
	Latency until the first hole visit	
Locomotion	Total number of line crossings	Line crossing with all its paws in the box
	Latency until the first line crossing	

However, previous studies using the mHB have shown that these separate behaviors scored in this assay can be reliably summarized to underlying behavioral dimensions; avoidance behavior, exploration and locomotion [51–53]. Two previous studies showed that simultaneous clustering of these behavioral dimensions yields distinct response types that are displayed by individuals of C, B6N and 129S2 [31,32].

For each dimension, observed behaviors indicative of that dimension were summarized to integrated behavioral z-scores according to the procedure described in Labots et al. [53] and van der Goot et al. [31]. In short, this entailed that behavioral variables measuring different aspects of the same behavioral dimension were normalized to z-scores, and combined to a single integrated z-score representing that dimension. Combination of z-scores was done by averaging them. For normalization of each separate variable, we used the pooled data (across all strains) as a reference group, as suggested by Labots et al. [53]. An overview of all included variables per dimension is listed in S1 Table.

### 2.6. Statistics

All analyses were conducted with R version 4.0.0 in R-Studio [54]. Linear mixed models (LMMs) were run using the package ‘nlme’ [55]. The package ‘kml3d’ was used for running a multivariate cluster analysis on longitudinal response trajectories [56]. All Figures were created with GraphPad Prism (GraphPad Prism version 7.04 for Windows, Graphpad Software, La Jolla, California USA, www.graphpad.com).

#### Experimental Phase 1

The total number of individuals included for behavioral analysis per strain was C (*n* = 59), B6N (*n* = 60) and 129S2 (*n* = 60). The behavioral variables obtained in this phase were used to characterize mice on their individual response type (Table 1). Additional analyses assessing between strain differences in behavioral scores, as well as the collected pCORT data were not reported in the present paper because they fall beyond the scope of the manuscript. These results will therefore be reported elsewhere.

The procedure described in section 4.5 yielded three trajectories of integrated residual z-scores for each individual mouse, one trajectory per behavioral dimension: avoidance behavior, exploration and locomotion. These three trajectories were fit with LMMs to control for potentially confounding factors. The resulting standardized Pearson residuals could then be used for a clustering procedure.

For each behavioral dimension, potentially confounding variables were controlled for by including strain and experimenter as fixed factors, and individual mouse, test group and test order as random factors in the model. The variable ‘trial’ was intentionally left out of the model because we wanted to maintain this information in the residuals so that we could assess habituation of individual mice over time. Models were run with an autoregressive correlation structure for continuous time covariates (corCAR1) from the ‘nlme’ package.

Model assumptions were assessed visually by inspecting the standardized residuals through QQ-plots, histograms and residual plots [57,58]. The dimension avoidance behavior was logarithmically transformed to achieve normality of the residuals. A square root transformation was applied on exploration and locomotion was rank transformed. Heteroscedasticity was avoided using the ‘varIdent’ variance structure function from the ‘nlme’ package, allowing different residual spread for each level of the categorical variables in our model [58]. The dimensions avoidance behavior, exploration and locomotion included a variance function for ‘Strain’.

The resulting standardized Pearson residual integrated z-score trajectories were subsequently analyzed with a multivariate k-means clustering procedure for longitudinal data, *kml3d* [56]. The settings and rationale for using this method have been described in detail in [31]. Furthermore, the settings used in the present manuscript are identical to a previous study [32].

As described, three response trajectories were included for each individual mouse: avoidance behavior, exploration and locomotion. These were clustered simultaneously to assess the occurrence of homogenous subgroups of mice that shared similar responses (between them) on all three behavioral dimensions. Prior to analysis, the gap statistic was applied to evaluate whether the data was perhaps best represented by a single cluster using the package ‘cluster’ [59]. This was not the case. The gap statistic compares the within-cluster sum-of-squares to a null reference distribution of the data, which is then equivalent to a single cluster [60], and as such gives an indication of whether it is appropriate to partition the data into clusters. The cluster analysis compiled 1000 iterations for each *k* clusters between 2 and 6, resulting in 5000 cluster solutions.

The optimal number of clusters was selected using the approach of Clustering Validity Indices (CVI’s) as suggested by Kryszczuk and Hurley [61] and adjusted by Wahl et al. [62]. All details of this procedure are described in [31]. After obtaining the optimal clustering solution, we applied a bootstrapping procedure to determine the stability of the identified clusters. 200 random samples (of *n* = 179) were drawn from the original data with replacement, meaning that a particular individual could occur multiple times in one sample. If our clusters were stable, applying the multivariate clustering procedure in these 200 random samples should reveal similar cluster structures [63]. Similarity in cluster composition between the original analysis and the bootstrapping, samples was established by the Jaccard Index. For each individual mouse, the number of times (out of 200 bootstrap samples) it retained its original cluster was determined using the following formula: *number of times in the same cluster/total number of bootstrapping samples*. The individual similarity indices were subsequently averaged across mice to determine the overall Jaccard similarity index for each cluster.

Finally, to characterize the resulting clusters, LMMs analyzed the differences in integrated behavioral z-scores across trials between clusters on each behavioral dimension. Model assumptions and settings were identical to the settings described above. Cluster and trial were included as fixed predictors, as well as their interaction. Individual mouse (ID) and slope (trial nested in ID) were included as random factors. The integrated behavioral z-score for locomotion was rank transformed to improve the residual distribution. A variance function (‘varIdent’) was applied for Cluster in the model for avoidance behavior, to avoid heteroscedasticity. The model for exploration included a variance function on trial, while locomotion included a variance function for the different trials within Cluster.

Main and interaction effects from all LMMs were derived using *F*-tests with corresponding *P* value (*P* < 0.05). Statistical significance of random effects was computed by means of likelihood ratio tests and reported as Chi Square values. Pairwise comparisons were conducted using the package ‘emmeans’ [64] to follow up on main or interaction effects. To reduce the probability of a Type I error due to multiple comparisons, the α was adjusted using a Dunn-Šidák correction in all *post hoc* tests [65]. S2 Table lists an overview of all corrected α-values used in this manuscript. All *post hoc* tests were summarized as beta-estimates and their corresponding standard error, *t* statistic and *P* values. Effect sizes for *post hoc* tests were reported as Cohen’s *d*, and obtained via the package ‘emmeans’ [64]. The guidelines provided by Wahlsten [66] were used to interpret the absolute values of Cohen’s *d* (|d|). This extensive review of various phenotypes suggested the following interpretation of effects for neurobehavioral mouse studies: small effect, |*d*| < 0.5; medium effect, 0.5 < |*d*| < 1.0; large effect, 1.0 < |*d*| < 1.5; very large effect, |*d*| > 1.5.

#### Experimental Phase 2

The results of phase 2 were first analyzed on the combined data of the balanced and the unbalanced pool (*n* = 96 individuals, 48 pairs, *n* = 16 pairs/strain). Whether pairs were balanced or unbalanced on individual response type was incorporated in the factor ‘pool’ (2 levels, balanced/unbalanced). This dataset, with considerable sample size and power, allowed us to analyze treatment and strain effects while asking whether incorporating the factor ‘pool’ (accounting for individual response type versus not accounting for this variation) in our analyses would explain part of the variance in our model. For each behavioral dimension, generalized linear models (GLMs) analyzed the effect of dexmedetomidine between strains on integrated behavioral z-scores. Treatment, strain, pool and experimenter were included as fixed factors, as well as all interactions. The factor ‘block’ (representing test day, see *section 1*.*4 Experimental protocol*) was included as a random factor without any interactions (as suggested by Festing [67]).

In addition, a second series of GLMs analyzed treatment and strain effects separately for the balanced pool (pairs of mice that were balanced on individual response type, *n* = 48, 24 pairs, *n* = 8/strain), and the unbalanced pool (pairs of mice that were not balanced on response type, *n* = 48, 24 pairs, *n* = 8/strain). For each pool, GLMs analyzed the effect of dexmedetomidine on integrated behavioral z-scores. For each behavioral dimension, treatment, strain and experimenter were included as fixed factors, as well as all interactions. The factor block was included as a random factor, without interactions. Model assumptions were assessed visually by inspecting the standardized residuals through QQ-plots, histograms and residual plots [57,58].

For all analyses in phase 2, the integrated behavioral z-score for exploration was logarithmically transformed and that of locomotion was rank transformed to improve the residual distribution. In addition, Cooks distance identified locomotion scores of 4 mice as influential–these were 2 individuals that had not displayed any line crossing, resulting in the maximum score for latency to first line crossing (300 seconds) and 2 individuals with a latency to first line crossing > 180 seconds. These observations were retained for analysis.

Main and interaction effects from all GLMs were derived using *F*-tests with corresponding *P* value (*P* < 0.05). Effect sizes for the GLMs are reported as partial eta squared (*η*_*p*_^*2*^) with 95% CI, using the following cut-off limits: small effect, *η*_*p*_^*2*^ ≤ 0.03; medium/moderate effect, 0.03 < *η*_*p*_^*2*^ < 0.10; large effect, 0.10 ≤ *η*_*p*_^*2*^ < 0.20; very large effect, *η*_*p*_^*2*^ ≥ 0.20 [68]. Pairwise comparisons were conducted using the package ‘emmeans’ [64] to follow up on main or interaction effects. To reduce the probability of a Type I error due to multiple comparisons, the α was adjusted using a Dunn-Šidák correction in all *post hoc* tests [65], see S2 Table. All *post hoc* tests were summarized as beta-estimates and their corresponding standard error, *z* statistic and *P* values. Effect sizes for *post hoc* tests were reported as Cohen’s *d*, and obtained via the package ‘emmeans’ [64]. The guidelines provided by Wahlsten [66] were again used to interpret the absolute values of Cohen’s *d* (|d|), see *“Experimental Phase 1”*.

## 3. Results

### 3.1. Cluster analysis

**Fig 3:**
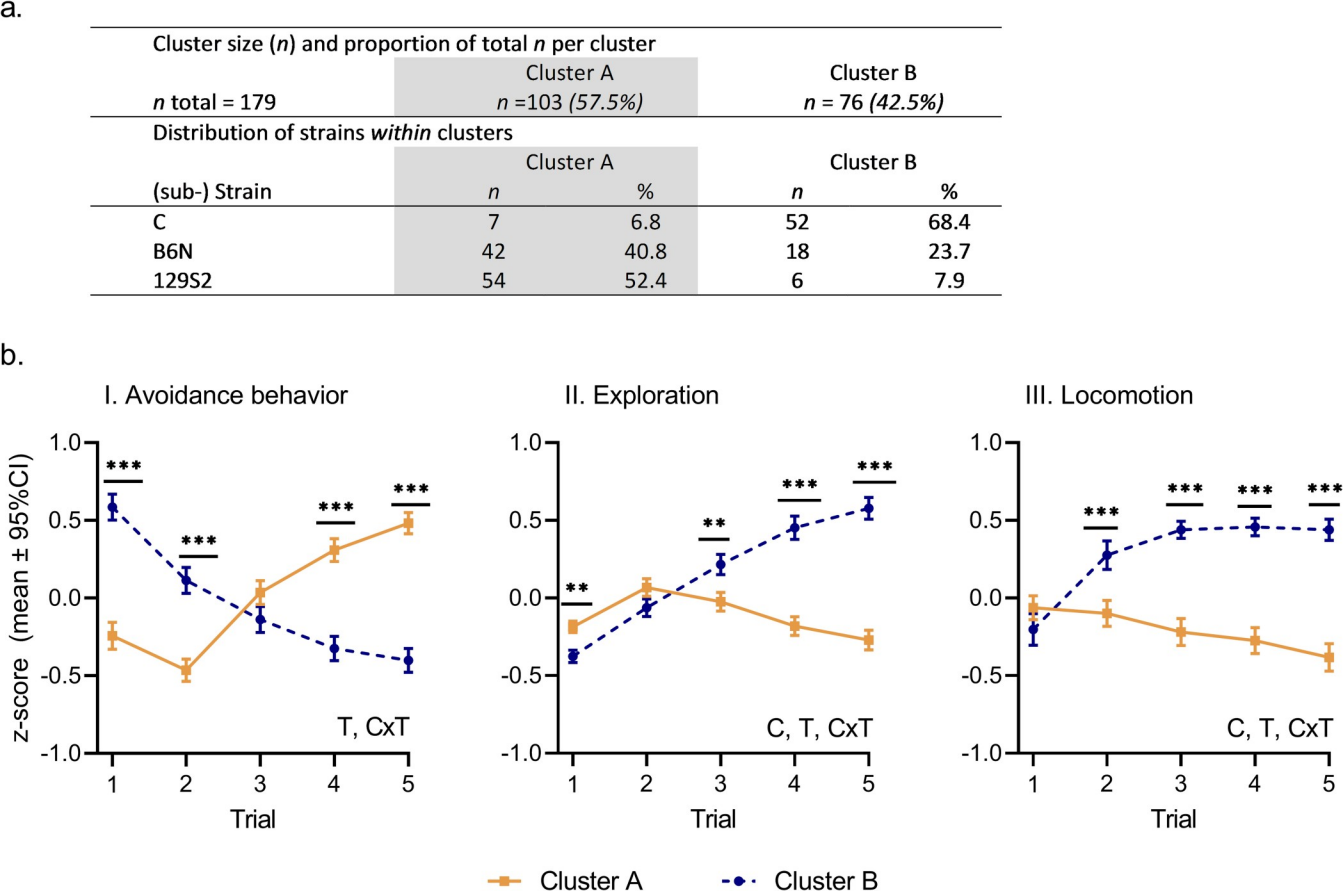
**(a)** Results cluster analysis. Top row: Cluster size and proportion of total population for each cluster (total number of mice, *n* = 179). Bottom rows: Distribution of strains (*n* and proportion) within each cluster. **(b)** Behavioral response trajectories of clusters on avoidance behavior, exploration and locomotion (cluster A, orange; cluster B, blue). Behavior expressed as integrated behavioral z-scores. Results are presented as means with 95% CI. Effects were significant in LMMs at *P* < 0.05. C: Significant main effect of cluster; T: Significant main effect of trial; C x T: Significant interaction between cluster and trial. Significant differences in *post hoc* contrasts between clusters on trials 1 and 5 (adjusted α = 0.025321) are indicated by ** = 0.00050 ≤ *P* < 0.00501, *** = *P* < 0.00050. Significant differences in *post hoc* contrasts between clusters on trials 2, 3 and 4 (α = 0.05) are indicated by ** = 0.001 ≤ *P* < 0.01, *** = *P* < 0.001. The raw scores (mean integrated behavioral z-score ± 95% CI) per trial, per cluster, for each behavioral dimension are listed in S3 Table.

The optimal partitioning of the data yielded two clusters, A and B. The table in Fig 3A presents cluster size and distribution of strains across clusters. The majority of individuals (57.5%, *n* = 103) grouped together in cluster A while the remaining mice formed cluster B (42.5%, *n* = 76). Each cluster consisted of mice from all three strains. The majority of C mice (88.1%, *n* = 52) grouped together in cluster B while the majority of 129S2 (90%, *n* = 54) and the majority of B6N (70%, *n* = 42) grouped together in cluster A. The clusters displayed differential patterns of behavior on all three behavioral dimensions, as indicated by significant interactions between clusters and trial (Avoidance behavior, trial effect: *F*_*(4*,*708)*_ = 13.17, *P* < 0.0001; interaction cluster x trial: *F*_*(4*,*708)*_ = 46.58, *P* < 0.0001; Exploration, cluster effect: *F*_*(1*,*177)*_ = 12.12, *P* = 0.0006; trial effect: *F*_*(4*,*708)*_ = 37.63, *P* < 0.0001; interaction cluster x trial: *F*_*(4*,*708)*_ = 44.97, *P* < 0.0001; Locomotion, cluster effect: *F*_*(1*,*177)*_ = 78.43, *P* < 0.0001; trial effect: *F*_*(4*,*708)*_ = 4.85, *P* = 0.0007; interaction cluster x trial: *F*_*(4*,*708)*_ = 19.64, *P* < 0.0001; Fig 3B).

*Post hoc* comparisons (adjusted α = 0.025320) showed that mice in cluster A increased avoidance behavior between the first and the last trial (-0.726 ± 0.96, *t*_*(708)*_ = -7.593, *P* < 0.0001, *large* effect size, *d* = -1.015, 95%CI [-1.283, -0.747]). At the same time, locomotion (rank transformed) decreased (98.06 ± 23.1, *t*_*(708)*_ = 4.250, *P* < 0.0001, *small* effect size, *d* = 0.498, 95%CI [0.267, 0.730]), while exploration remained stable across trials (0.085 ± 0.05, *t*_*(708)*_ = 1.583, not significant, S4-I Table).

Mice in cluster B displayed the opposite pattern and decreased avoidance behavior between the first and the last trial (0.985 ± 0.96, *t*_*(708)*_ = 10.288, *P* < 0.0001, *large* effect size, *d* = 1.377, 95%CI [1.105, 1.650]), while exploration and locomotion increased (exploration, -0.953 ± 0.06, *t*_*(708)*_ = -15.274, *P* < 0.0001, *very large* effect size, *d* = -3.588, 95%CI [-4.085, -3.090]; locomotion (rank transformed), -252.68 ± 32.6, *t*_*(708)*_ = -7.740, *P* < 0.0001, *large* effect size, *d* = -1.285, 95%CI [-1.618, -0.952]), see S4-I Table.

The trajectories of avoidance behavior were significantly higher in cluster B on the first two trials (trial 1, *P* < 0.0001, *large* effect size, *d* = -1.158, 95%CI [-1.488, -0.827]; trial 2, *P* < 0.0001, *medium* effect size, *d* = -0.808, 95%CI [-1.127, -0.489]) and lower than cluster A on trials 4 and 5 (trial 4, *medium* effect size, *P* < 0.0001, *d* = 0.887, 95%CI [0.565, 1.208]; trial 5, *large* effect size, *P* < 0.0001, *d* = 1.235, 95%CI [0.901, 1.569], Fig 3B-I and S4-II Table). Furthermore, exploration in cluster B was lower on trial 1 (*P* = 0.0011, *medium* effect size, *d* = 0.715, 95%CI [0.283, 1.147]) and higher on the last three trials (trial 3, *P* = 0.0042, *medium* effect size, *d* = -0.903, 95%CI [-1.525, -0.281]; trial 4, *P* < 0.0001, *very large* effect size, *d* = -2.384, 95%CI [-3.135, -1.635]; trial 5, *P* < 0.0001, *very large* effect size, *d* = -3.192, 95%CI [-4.009, -2.375], Fig 3B-II and S4-II Table). Locomotion was significantly higher in cluster B on all trials except trial 1 (trial 2, *P* < 0.0001, *medium* effect size, *d* = -0.774, 95%CI [-1.140, -0.406]; trial 3, *P* < 0.0001, *large* effect size, *d* = -1.157, 95%CI [-1.510, -0.798]; trial 4, *P* < 0.0001, *large* effect size, *d* = -1.384, 95%CI [-1.730, -1.033]; trial 5, *P* < 0.0001, *very large* effect size, *d* = -1.562, 95%CI [-1.910, -1.208], Fig 3B-III and S4-II Table).

#### 3.1.1 Cluster stability

**Fig 4:**
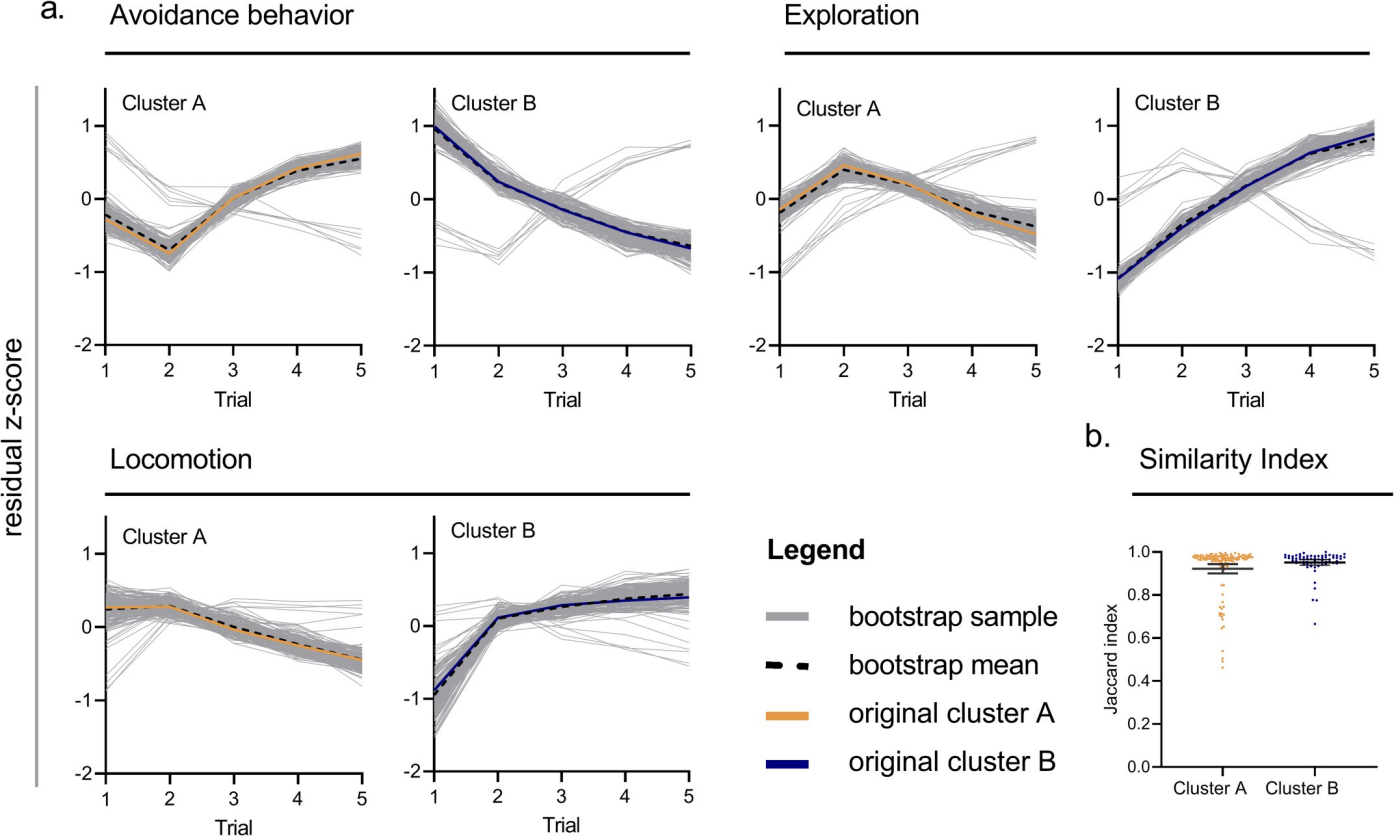
**(a)** Results of the bootstrapping procedure for each cluster, on each behavioral dimension. Results are presented as mean residual z-scores, and depict the trajectory of the original cluster (cluster A, orange; cluster B, blue) in relation to the average of all 200 bootstrap samples (black dashed line), against all 200 trajectories of the bootstrapping procedure (grey). **(b)** Distribution of individual Jaccard Indices in clusters A and B (cluster A, orange dots; cluster B, blue dots). Average Jaccard Index per cluster indicated by mean with 95% CI (black).

Cluster stability was assessed with a bootstrapping procedure in which 200 random samples (of *n* = 179) were drawn from the original data with replacement. The trajectories of the original cluster and the average of all 200 cluster analyses were highly similar (Fig 4A).

The average Jaccard Index was 0.92 for cluster A (Fig 4B), meaning that on average, mice that fell in cluster A also did so in 92% of the bootstrap samples. The average Jaccard Index for cluster B was equally high (0.95, Fig 4B). The originally obtained clusters A and B were thus rendered stable, and representative for this dataset.

### 3.2. Results pharmacological experiment

The cluster analysis revealed two differential response types, which were displayed by individuals of all three strains. We next explored whether incorporating these individual response types in the design of a standard pharmacological experiment would affect the results in comparison to an experiment in which this variation was not controlled for. A 2 (treatment) x 3 (strain) x 2 (experimenter) factorial complete randomized block design was used to test the effect of dexmedetomidine on behavior in the mHB.

#### 3.2.1 Incorporating individual variation as a discriminating factor in analysis

The results were first analyzed on the total population (*n* = 96, 48 pairs, *n* = 16 pairs/strain), the combined data of the unbalanced and the balanced pool. Generalized linear models (GLMs) analyzed the effect of dexmedetomidine on behavior using a 2 (treatment) x 3 (strain) x 2 (pool) x 2 (experimenter) factorial design, including all interactions. The factor ‘block’ (test day, *n* = 4) was included as a random factor without any interactions [67].

**Fig 5:**
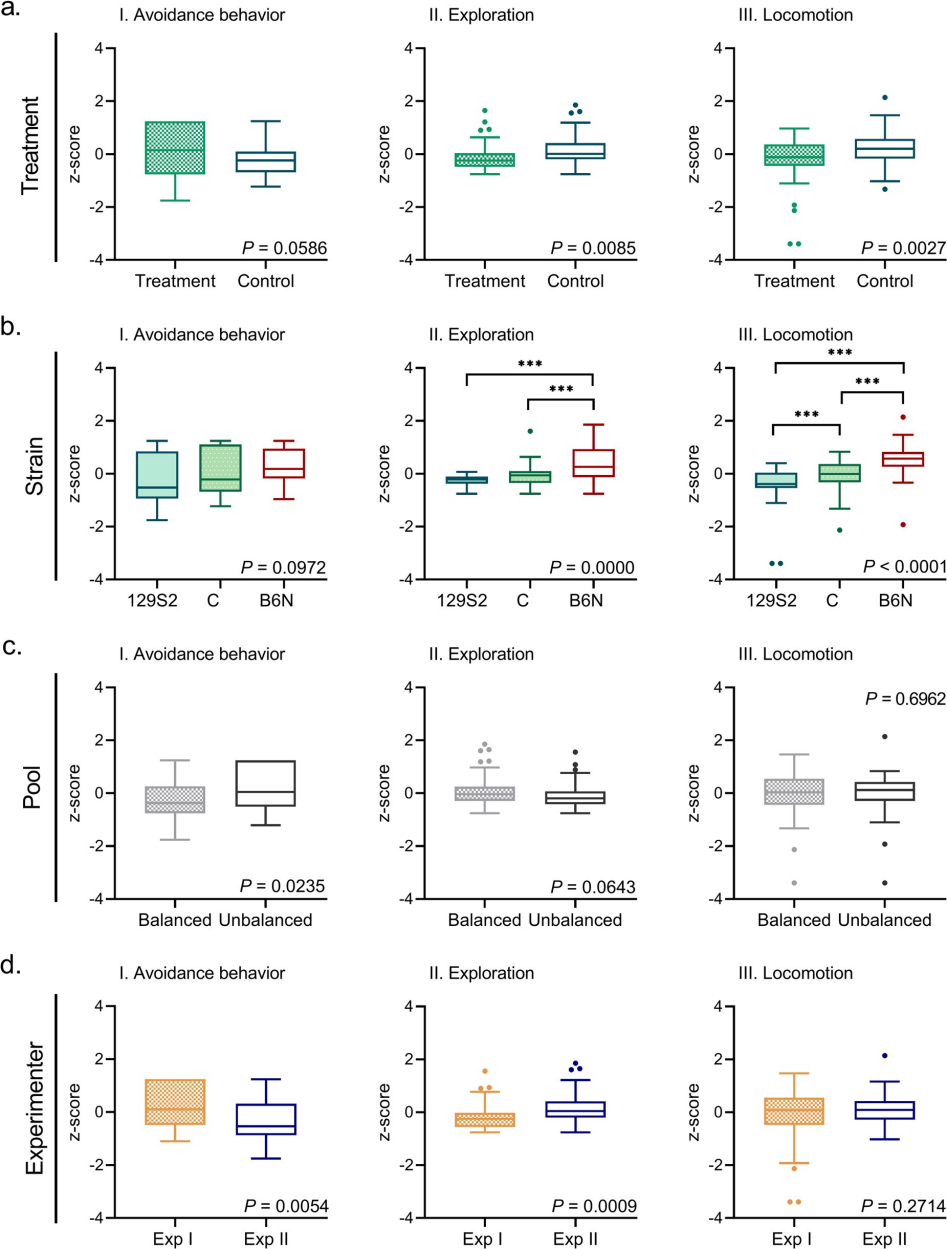
**(a-d)** Avoidance behavior, exploration and locomotion of mice in a single 5-minute mHB trial. Behavior in all graphs expressed as integrated behavioral z-score. Results are presented as boxplots (median, upper and lower quartiles) with Tukey whiskers. Individual points = outside the upper/lower quartile*1.5 inter-quartile range. Effects were significant in GLMs at *P* < 0.05. Significant differences in *post hoc* contrasts between strains (adjusted α = 0.02532) are indicated by *** when *P* < 0.00050. The raw integrated z-scores (mean ± 95% CI) of each group depicted in this figure are presented in S5 Table.

Treatment with dexmedetomidine primarily reduced activity related behavior, compared to a control injection with saline. Treated mice displayed less exploration (*F*_*(1*,*68)*_ = 7.36, *P* = 0.0085, *medium* effect size, *η*_*P*_^*2*^ = 0.097, 95% CI [0.008, 0.252]) and less locomotion than controls (*F*_*(1*,*68)*_ = 9.75, *P* = 0.0027, *large* effect size, *η*_*P*_^*2*^ = 0.124, 95% CI [0.016, 0.279]; Fig 5A-II and 5A-III) and S5 Table). The effect of dexmedetomidine on anxiety related behavior was less pronounced, but there was a suggestion of average higher levels of avoidance behavior in treated animals (F_(1,68)_ = 3.70, *P* = 0.0586, *medium* effect size, *η*_*P*_^*2*^ = 0.052, 95% CI [0.000, 0.185]; Fig 5A-I and S5 Table).

Strain also differed significantly in exploration (*F*_*(2*,*68)*_ = 13.38, *P* = 0.0000) and locomotion (*F*_*(2*,*68)*_ = 29.23, *P* < 0.0001), both with very large effect sizes (respectively *η*_*P*_^*2*^ = 0.282, 95%CI [0.104, 0.423]; *η*_*P*_^*2*^ = 0.493, 95%CI [0.319, 0.616]; Fig 5B-II and 5B-III and S5 Table).

*Post hoc* comparisons (adjusted α = 0.02532) showed that exploration was highest in B6N compared to C and 129S2 (C, *P* = 0.0002, *medium* effect size, *d* = -0.928, 95%CI [-1.499, -0.408]; 129S2, *P* < 0.0001, *large* effect size, *d* = -1.327, 95%CI [-1.891, -0.763]; Fig 5B-II and S6A Table). Furthermore, locomotion differed between all three strains, with higher levels of locomotion in B6N compared to C and 129S2 (C, *P* < 0.0001, *large* effect size, *d* = -1.240, 95%CI [-1.780, -0.700]; 129S2, *P* < 0.0001, *very large* effect size, *d* = -2.000, 95%CI [-2.620, -1.381]) and higher locomotion in C than in 129S2 (*P* = 0.0028, *medium* effect size, *d* = -0.760, 95%CI [-1.270, -0.246], Fig 5B-III and S6A Table).

Avoidance behavior did not significantly differ between strains (*F*_*(2*,*68)*_ = 2.41, *P* = 0.0972, medium effect size, *η*_*P*_^*2*^ = 0.068, 95% CI [0.000, 0.227]). A significant effect of pool however, demonstrated that avoidance behavior was significantly lower in pairs that were matched on response type, than in pairs that were not matched on response type (*F*_*(1*,*68)*_ = 5.37, *P* = 0.0235, *medium* effect size, *η*_*P*_^*2*^ = 0.074, 95%CI [0.000, 0.209]; Fig 5C-I and S5 Table). A suggestion of a similar effect (in reversed direction) was found for exploration (*F*_*(1*,*68)*_ = 3.54, *P* = 0.0643, medium effect size, *η*_*P*_^*2*^ = 0.049, 95%CI [0.000, 0.175]; Fig 5C-II and S5 Table).

The results also revealed experimenter effects for avoidance behavior and exploration: Experimenter I scored higher levels of avoidance behavior and lower levels of exploration than Experimenter II (Avoidance behavior, *F*_*(1*,*68)*_ = 8.26, *P* = 0.0054, *large* effect size, *η*_*P*_^*2*^ = 0.106, 95%CI [0.010, 0.254]; Exploration, *F*_*(1*,*68)*_ = 11.95, *P* = 0.0009, *large* effect size, *η*_*P*_^*2*^ = 0.150, 95%CI [0.027, 0.301]; Fig 5D-I and 5D-II and S5 Table).

All in all, these results indicate that behavioral scores may differ between an experimental pool in which individual differences are accounted for, and a pool in which this variation is not incorporated (such as for avoidance behavior). The absence of any significant interactions however suggest that this effect of pool did not interfere directly with treatment or strain effects.

#### 3.2.2 Comparison between balanced and unbalanced pool

Next, we analyzed the balanced and the unbalanced pool separately and compared the results. Aside for controlling for individual response type, we considered the two pools directly comparable with respect to other factors such as experimenter, strain, treatment etcetera. Any difference in results between these two pools of mice was therefore attributed to the fact that we matched our pairs on individual response type in one pool (balanced) and not in the other (unbalanced).

Furthermore, following the principle of good experimental design described in the introduction, matching our pairs on individual response types in the balanced pool should make the treatment and control groups more similar, thereby improving the quality of our results. Any differences in results between the balanced and the unbalanced pool were thus interpreted in favor of the balanced data.

For each pool, GLMs analyzed the effect of dexmedetomidine on behavior using a 2 (treatment) x 3 (strain) x 2 (experimenter) factorial design, including all interactions. The factor ‘block’ (test day, *n* = 4) was included as a random factor without any interactions [67].

**Fig 6:**
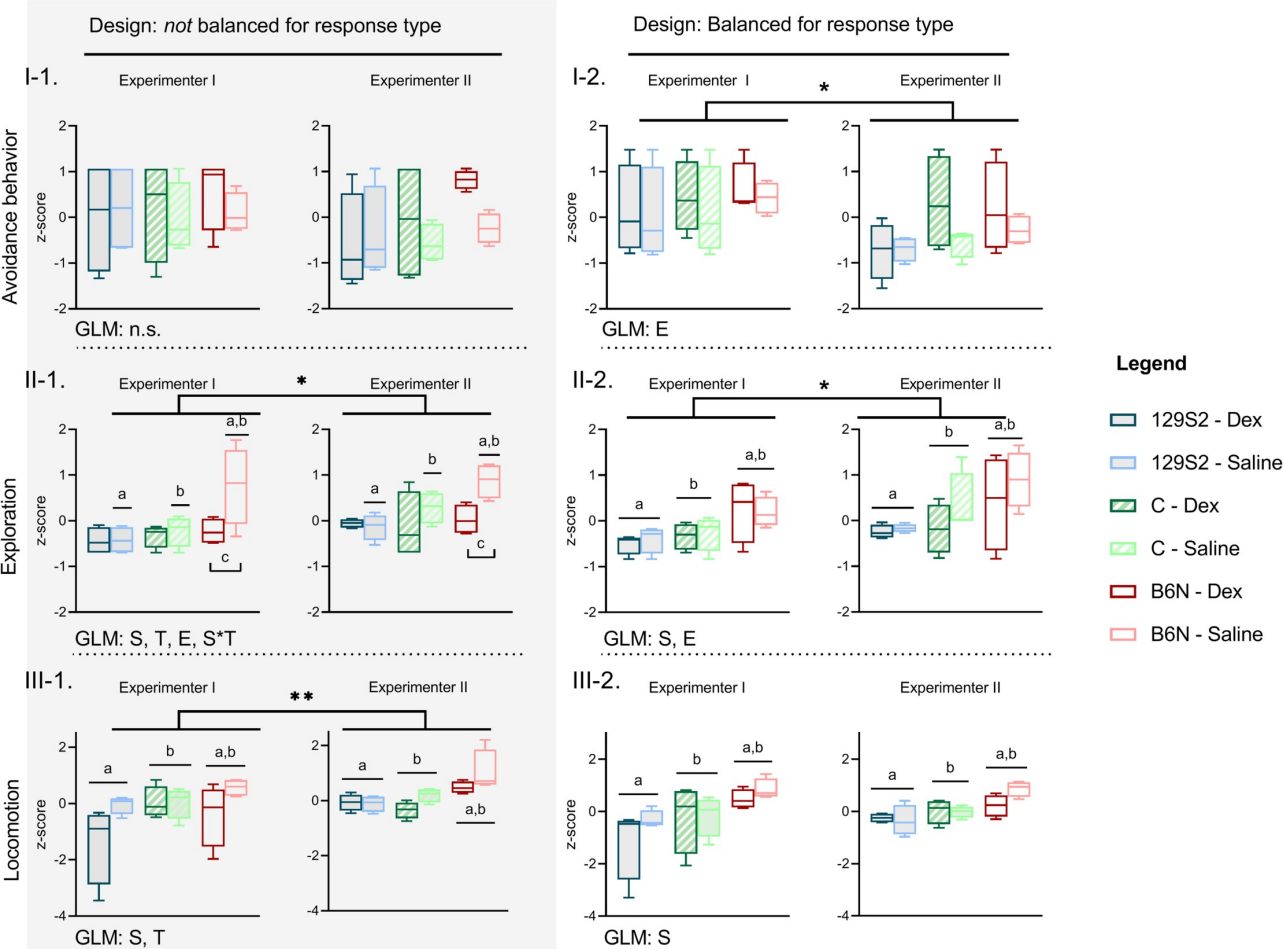
Behavioral scores of a pool of mice not balanced for individual response type (left), and a pool balanced for individual response type (right). **(I-III)** Results expressed as integrated behavioral z-scores and presented as boxplots (median, upper and lower quartiles) with Tukey whiskers. Effects significant in GLMs at *P* < 0.05, where * = 0.01 ≤ *P* < 0.05, ** = 0.001 ≤ *P* < 0.01. T: Significant effect treatment; S: Significant effect strain; S*T: Significant interaction between treatment and strain; E: Significant effect experimenter; n.s. = no significant difference. Significant *post hoc* comparisons indicated with a lower-case letter, where **(II-1)** the same lower-case letter above two single boxplots indicates a significant *post hoc* comparison between strains for one treatment condition only and **(II-2, III-1, III-2)** the same lower-case letter above combined boxplots indicates a significant *post hoc* comparison between strains regardless of treatment (*P*-value adjusted for multiple comparisons, S2 Table). The raw integrated z-scores (mean ± 95% CI) of each group depicted in this figure are presented in S7 Table.

Separate analyses of the balanced and the unbalanced pool indeed yielded different results, especially with respect to exploration and locomotion. In the unbalanced pool, treatment effects on exploration differed between strains (strain effect: *F*_*(2*,*32)*_ = 9.17, *P* = 0.0007, *very large* effect size, *η*_*P*_^*2*^ = 0.364, 95% CI [0.088, 0.538]; treatment effect: *F*_*(1*,*32)*_ = 9.13, *P* = 0.0049, *very large* effect size, *η*_*P*_^*2*^ = 0.222, 95%CI [0.023, 0.433]; interaction strain x treatment: *F*_*(2*,*32)*_ = 4.98, *P* = 0.0131, *very large* effect size, *η*_*P*_^*2*^ = 0.238, 95%CI [0.000, 0.428]; Fig 6II-1 and S7 Table).

*Post hoc* comparisons between strains in each condition (adjusted *α* = 0.01274) revealed that exploration in the unbalanced pool only differed between strains in the control groups: saline injected B6N displayed more exploration than saline injected C and 129S2 (C, *P* = 0.0002; *very large* effect size, *d* = -1.901, 95%CI [-2.997, -0.805]; 129S2, *P* < 0.0001, *very large* effect size, *d* = -2.531, 95%CI [-3.691, -1.371]; Fig 6II-1 and S6C Table).

Furthermore, *post hoc* comparisons between conditions within strain (adjusted *α* = 0.016952) showed that saline injected B6N displayed more exploration than their counterparts treated with dexmedetomidine (*P* < 0.0001, *very large* effect size, *d* = 2.105, 95%CI [0.997, 3.213]; Fig 6II-1 and S6C Table).

Interestingly, this treatment effect disappeared when analyzing the balanced pool. Exploration still differed between strains (strain effect: *F*_*(2*,*32)*_ = 8.24, *P* = 0.0013, *very large* effect size, *η*_*P*_^*2*^ = 0.340, 95%CI [0.070, 0.518]) but exploration was now higher in B6N than C and 129S2 regardless of treatment, as opposed to only in the saline condition (C, *P* = 0.0031; *large* effect size, *d* = -1.053, 95%CI [-1.800, -0.310]; 129S2, *P* = 0.0001, *very large* effect size, *d* = -1.719, 95%CI [-2.690, -0.743]; Fig 6II-2 and S6B Table). Also, the treatment effect within B6N disappeared in the balanced condition (Fig 6II-2).

A similar shift of the effect of treatment was found for locomotion. In the unbalanced pool, locomotion was significantly higher in controls compared to treated mice across strains (*F*_*(1*,*32)*_ = 10.15, *P* = 0.0032, *very large* effect size, *η*_*P*_^*2*^ = 0.241, 95%CI [0.032, 0.450]; Fig 6III-1 and S7 Table) but when controlling for individual response type this treatment effect disappeared in the balanced pool (*F*_*(1*,*32)*_ = 1.62, *P* = 0.2224, *medium* effect size, *η*_*P*_^*2*^ = 0.046, 95%CI [0.000, 0.249]; Fig 6III-2 and S7 Table).

Similar to exploration, strains differed in locomotion in both the balanced (*F*_*(2*,*32)*_ = 13.78, *P* = 0.0002, *very large* effect size, *η*_*P*_^*2*^ = 0.463, 95%CI [0.176, 0.616]; Fig 6C-II) and the unbalanced pool (*F*_*(2*,*32)*_ = 12.05, *P* = 0.0001, *very large* effect size, *η*_*P*_^*2*^ = 0.430, 95%CI [0.144, 0.590]; Fig 6III-1). *Post hoc* comparisons (adjusted α = 0.02532) showed that locomotion (rank transformed) was significantly higher in B6N than in C and 129S2 in both the unbalanced (C, *P* = 0.0021, *large* effect size, *d* = -1.116, 95%CI [-1.880, -0.354]; 129S2, *P* < 0.0001, *very large* effect size, *d* = -1.713, 95%CI [-2.520, -0.903]; Fig 6C-I and S6C Table) and balanced condition (C, *P* = 0.0002, *large* effect size, *d* = -1.320, 95%CI [-2.090, -0.520]; 129S2, *P* < 0.0001, *very large* effect size, *d* = -2.250-, 95%CI [-3.290, -1.209], Fig 6III-2 and S6B Table).

In contrast to activity related behavior, avoidance behavior was not affected by treatment in both the unbalanced (*F*_*(1*,*32)*_ = 1.33, *P* = 0.2573, *medium* effect size, *η*_*P*_^*2*^ = 0.040, 95%CI [0.000, 0.023]; Fig 6I-1) and the balanced pool (*F*_*(1*,*32)*_ = 2.18, *P* = 0.1499, *medium* effect size, *η*_*P*_^*2*^ = 0.064, 95%CI [0.000, 0.277]; Fig 6I-2). Also, strains did not differ in avoidance behavior in either pool (unbalanced, *F*_*(2*,*32)*_ = 1.31, *P* = 0.2830, *medium* effect size, *η*_*P*_^*2*^ = 0.076, 95%CI [0.000, 0.272]; balanced, *F*_*(2*,*32)*_ = 0.64, *P* = 0.5349, *medium* effect size, *η*_*P*_^*2*^ = 0.038, 95%CI [0.000, 0.339]; Fig 6I-1 and 6II-2 and S7 Table).

The differences in results for exploration and locomotion show that the variation related to individual response types may augment observed treatment effects, as these effects disappeared when this variation was controlled for. Analysis of avoidance behavior in the unbalanced and the balanced pool however suggests that variation related to individual response type may also exert an opposite effect, in the sense that it may mask variation related to confounding factors. In the unbalanced pool, observed levels of avoidance behavior were not significantly different between Experimenters I and II (*F*_*(1*,*32)*_ = 1.86, *P* = 0.1825, *medium* effect size, *η*_*P*_^*2*^ = 0.055, 95%CI [0.012, 0.252]; Fig 6I-1 and S7 Table). Controlling for individual response type in the balanced condition however, resulted in significantly higher levels of observed avoidance behavior for Experimenter I than for Experimenter II (*F*_*(1*,*32)*_ = 7.35, *P* = 0.0107, *large* effect size, η_P_^2^ = 0.180, 95%CI [0.011, 0.399]; Fig 6I-2 and S7 Table). Experimenter effects were also found for exploratory activity, but now in both pools: Observed levels of exploration were higher in Experimenter II than in Experimenter I in the unbalanced pool (*F*_*(1*,*32)*_ = 6.56, *P* = 0.0154, *large* effect size, *η*_*P*_^*2*^ = 0.168, 95%CI [0.006, 0.384]; Fig 6II-1 and S7 Table) and the balanced pool (*F*_*(1*,*32)*_ = 5.29, *P* = 0.0281, *large* effect size, *η*_*P*_^*2*^ = 0.135, 95%CI [0.000, 0.356]; Fig 6II-1 and S7 Table).

## 4. Discussion

Matching experimental animals on their individual response type in control and test groups yielded different results than a comparable experimental pool in which these response types were not accounted for. These results demonstrate how including inter-individual variability in the composition of experimental groups may alter the observed pharmacological effects on behavioral performance. Also, by directly comparing a design in which inter-individual variability was accounted for (the balanced pool) versus a design in which this was not accounted for (the unbalanced pool) this study, to our knowledge, is the first to empirically demonstrate how this variability indeed may affect experimental outcomes.

As noted in the Introduction, the demonstration of a confounding effect of inter-individual variability in itself is not new, as several examples exist of how sub-populations within an experimental pool may mask the detection of overall group effects (i.e. increase the risk of a Type II error [14,19]. Such a masking effect was confirmed in the present experiment where matching individuals on their response type revealed a confounding experimenter effect for avoidance behavior that was not observed in the opposing design in which inter-individual variability was not accounted for. Also, inter-individual variability appeared to augment treatment effects for activity behavior (Fig 6). These results thus support the claim that a more active consideration of inter-individual variability in the design and outcomes of neurobehavioral preclinical research could contribute to the quality and reproducibility of experimental results [3].

In addition, this study demonstrated how data-driven analysis techniques, such as the clustering approach applied here, may facilitate an individual-based characterization of behavioral responses that encompasses the entire spectrum of variability in our data. As outlined in the Introduction, the advantage of such a data-driven approach in animal models of behavioral dysfunction is that it more closely matches the conceptualization of human psychopathology on a continuous spectrum [26]. Thereby it provides a more refined alternative to other strategies that harness inter-individual variability by separating subpopulations based on a predefined criterion [14]. The present study also provided a methodological example of how inter-individual variability may be considered as a variable in the design of animal experiments. Interestingly, a highly similar approach was recently demonstrated by Rojas-Carvajal et al. [12], who first characterized unconditioned responses to novelty in individual Sprague-Dawley and Wistar outbred rats and subsequently used this information to balance experimental groups such that inter-individual variability in the read-out parameter of interest was equally distributed within and between experimental groups. This study, together with the one presented here, demonstrates how a priori characterization of individual experimental animals may enable researchers to actively control for any inter-individual variability in the design of their experiments.

At the same time however, we recognize that the procedure of a priori characterization may not be suitable or desirable for all phenotypes or contexts. In behavioral neurosciences for example, initial testing for the purpose of individual characterization may be undesirable because the animals need to be naïve to the test, although evidence suggests that naivety to the test can be ensured by allowing sufficient time between characterization and test [68,69]. Second, characterizing individuals and subsequently using this characterization in experimental design requires that the observed trait is consistent across time. With respect to anxiety, individuality in both anxiety-related and activity behaviors have indeed often been shown to be repeatable and consistent through time and context in isogenic mice [27–29,70–72]. Other behavioral traits however, such as grooming behavior, have been shown to be less consistent across time within the same individual [72]. In the present study we unfortunately did not test the temporal consistency of our individual response types. The identified clusters were stable however at the time of assessment (Fig 2) and the behavioral profiles of the response types largely overlapped with two previous studies [31,32], suggesting some consistency. This aspect requires further validation, as confirmation of temporal stability of our response types will substantiate our claim that the difference in results between a balanced and the unbalanced pool can be attributed to variation related to inter-individual differences in response.

Third, for some phenotypes, a priori characterization may simply not be possible because this phenotype is only expressed during a limited time window, such as the isolation calling response, or ultrasonic vocalization in rodent pups [73], or social play behavior in rats [74]. And, lastly, a priori characterization may present some serious challenges from a time and cost perspective. On that note, it should be emphasized that some of the more labor-intensive aspects of the present study (multiple trials for characterization of the animals, scoring multiple behavioral categories) were tied specifically to our phenotype of interest: temporal change of anxiety and activity related behavior. Other paradigms, assessing less complex phenotypes could suffice with less complex ethograms, a single trial, or even assessment in an automated home cage environment prior to testing.

A highly efficient alternative to a priori characterization is the use of cross-over designs [75,76]. In these designs, for example a balanced Latin Square design, variability between individuals is accounted for by contrasting all treatments within the same animal [76]. Each animal thereby serves as its own control, which reduces the sample size while maintaining the same statistical power [76]. With respect to the current study, it should be noted that this would indeed have been a more practical and time efficient strategy to account for inter-individual variability if evaluating the effectiveness of dexmedetomidine as an anxiolytic would have been the sole purpose of this paper. As described above however, our primary aim was not so much to study dexmedetomidine as an anxiolytic, but to evaluate whether balancing experimental groups with respect to inter-individual variability would yield different results compared to a ‘regular’ drug study in which this variability is not actively accounted for.

Also, analogous to the strategy of a priori characterization, cross-over designs may not be suitable for all research contexts. A prerequisite for cross-over designs for example is that order effects are controlled for by balancing test order between subjects and that the applied treatment does not permanently alter the subjects [76]. In addition, similar to our approach, a sufficient wash-out period should be allowed between treatments to avoid carry-over effects [76]. Due to these requirements, cross-over designs are less suitable in studies that address the brain-behavior relationship in response to one or multiple compounds, as is common in research addressing the neurobiological underpinnings of behavioral dysfunction [77]. Similarly, these designs are less suitable in studies that evaluate chronic drug treatment, for example in the context of depression and anxiety [78,79]. All in all, both methods have their strengths in terms of controlling for inter-individual variability and what method is preferred highly depends on the research objective.

In addition to accounting for inter-individual variability in experimental design, one may also account for individual responses in statistical analysis of the results. In the present study, we used LMMs to analyze cluster differences and the effects of dexmedetomidine because these techniques have been proven especially effective in accounting for between- and within-individual variability [80]. The advantage of these models is that multiple characteristics of individual response (i.e. variability between and/or within individuals, differences in residual variation among individuals) can be accounted for in a single model [80,81]. LMMs also present a number of advantages over traditional analysis of variance (ANOVA/ANCOVA) when random effects are present, such as increased power, flexibility towards non-normally distributed data and the ability to handle missing values [82]. Accounting for such random effects in addition reduces the probability of false positives (Type I error rates) and false negatives (Type II error rate, [83]).

Next, in addition to these methodological and statistical considerations, this study confirmed previously identified inter-individual differences in the ability to habituate anxiety-related behavior in C, 129S2 and B6N [32]. The profiles were characterized by differential patterns of avoidance behavior and exploration: Mice in Cluster A combined an increase of avoidance behavior with low levels of exploration that remained stable over trials, while avoidance behavior decreased and exploration increased in Cluster B (Fig 3). This interplay between anxiety and exploration may be explained by the so-called approach-avoidance conflict that exposure to novel environments may induce in rodents, which entails the motivational conflict between the drive to explore a novel environment and the motivation to avoid potentially harmful stimuli [13,33,34]. Following this interplay, the decrease in avoidance behavior in Cluster B may be interpreted as successful habituation of anxiety responses, while the increase in avoidance in Cluster A may be considered as impaired habituation to the test.

In addition to contrasting patterns of avoidance behavior and exploration however, the clusters were characterized by significant differences in locomotor activity. The low levels of locomotion that decreased over trials in Cluster A contrasted with the pronounced increase in locomotion in Cluster B. Locomotion is not only associated with general activity levels, but differences in locomotion may also confound the interpretation of avoidance behavior as an indicator of anxiety, as the lack of exploration of an unprotected area may just as well be the result of reduced activity [33,34]. Whether the two individual response types reflect differential anxiety-phenotypes, or whether they merely reflect differential activity levels requires further study. A more elaborate discussion on this matter, along with suggestions for future research is provided in [32].

Furthermore, this study presented marked experimenter differences in behavioral scores for avoidance behavior and exploration, despite the fact that the experiment was carefully balanced with respect to experimenter. Behavioral phenotyping of anxiety-related behavior in rodents has indeed been demonstrated to be sensitive to experimenter-related factors such as handling style and familiarity with the experimenter [84,85]. In this study, both experimenters were naïve to handling rodents and were trained by the same person to handle the mice. Furthermore, all animals were handled by both experimenters from arrival at the test facility onwards. These measures however unfortunately do not preclude the possibility that handling differences or other experimenter-related factors affected the outcome of this study as experimenters themselves can never be entirely subjected to standardization [86]. Another experimenter-induced factor that may affect the scoring of live behavior is observer variability [38,87,88]. Both inter- and intra-observer reliability were established at a good to excellent level prior to the start of the study, after a training phase in which both experimenters aligned coding by scoring video data from previously collected mHB-data. Observer reliability in itself may however again be affected by coding experience, rapidity of the behavior, energy level of the observer and so on [11,89]. These factors illustrate how complete control over experimenter-induced variability is difficult to accomplish. In fact, the experimenter has been termed one of the most uncontrollable background factors in experimental research, affecting experimental outcomes and reproducibility between studies in a similar manner as inter-individual variability [38].

Automated tracking may form a way to overcome this incontrollable nature and as such to increase the standardization of an experiment [88]. Fully automated scoring has unfortunately not yet been validated in the modified Hole Board however, and doing so was beyond the scope of the present study. It has been suggested however that experimenter effects should not greatly reduce the power to detect treatment effects provided the experiment was carefully balanced for the inclusion of multiple experimenters, and experimenter is included as a factor in the data analysis [87]–which was indeed the case in the present study. Also, systematic incorporation of multiple experimenters was recently suggested by Richter [38] as a means to account for potentially confounding experimenter-induced variation. This concept, termed systematic heterogenization, entails that one may improve the generalizability of results by systematically incorporating known sources of experimental variation (such as experimenter) in the design of a single experiment [8].

Finally, this study does not provide definitive conclusions about the potential of dexmedetomidine as an anxiolytic. Treatment with dexmedetomidine resulted in (a suggestion of) higher anxiety related behavior in treated animals, while exploration was significantly lower. This could be interpreted as anxiogenic according to the aforementioned interplay between anxiety-related behavior and exploration. We suspect however that the observed effects may rather have been associated with sedation, because locomotor activity was significantly lower in treated animals. Previous studies confirm a sedative effect of dexmedetomidine, as indicated by reduced locomotor activity [90,91]. Our choice for keeping the dose of dexmedetomidine constant across strains was motivated by our objective to keep factors other than individual response type the same between test groups. For a correct evaluation of the effect of dexmedetomidine however, inspecting strain-dependent dose-response behaviors would have probably been more appropriate as different mouse strains have demonstrated differences in a2-adrenergic receptor-binding [92,93]. This anxiogenic/sedative effect however did not interfere with the main objective of this paper.

## Conclusions

This study empirically demonstrated that inter-individual variability may mask or augment experimental results. In addition, it provides an example approach of how this variability can be incorporated in experimental design, and how phenotypes that rely on the temporal nature of a response may be defined on an individual, multivariate level. As such it contributes to the existing literature that explores new approaches and viewpoints in experimental design and analysis with the goal to improve the quality and reproducibility of experimental results.

## Supporting information

S1 Table Behavioral variables measured in mHB and used for composition of z-scores in this paper. (DOCX)

S2 Table Overview of Dunn-Sidak corrected values for α in *post hoc* comparisons. (DOCX)

S3 Table Mean integrated behavioral z-score and corresponding 95% confidence interval for each cluster (A/B) on each trial (1–5) for avoidance behavior, exploration and locomotion.
(DOCX)

S4 Table Post hoc tests comparing either (I) the estimated marginal means between trials 1 and 5 (adjusted α = 0.016952) for avoidance behavior, exploration and locomotion and (II) cluster differences on each trial for avoidance behavior, exploration and locomotion (adjusted α = 0.016952). Significant comparisons are highlighted in bold.
(DOCX)

S5 Table Raw integrated z-scores (mean ± 95% confidence interval) of groups (n = 8/group) that were compared in GLMM’s to test the effects of treatment, strain, pool and experimenter on avoidance behavior, exploration and locomotor activity, using a 2 (treatment) x 3 (strain) x 2 (experimenter) x 2 (balanced/unbalanced pool) factorial design, including all interactions. (DOCX)

S6 Table Post hoc tests comparing (a) the estimated marginal means between strains (adjusted α = 0.025321) for each behavioral dimension, on the total dataset, so balanced and unbalanced combined, section 2.2.1 (b) strain differences on each behavioral dimension(adjusted α = 0.025321) for the balanced data only and (c) strain differences on avoidance behavior and locomotion (adjusted α = 0.025321) or strain comparisons within treatment/within strain comparisons between treatments (adjusted α = 0.016952) for exploration. Significant comparisons are highlighted in bold.
(DOCX)

S7 Table Raw integrated z-scores (mean ± 95% confidence interval) of groups (*n* = 4/group) that were compared in GLMM’s to test the effects of treatment, strain, pool and experimenter on avoidance behavior, exploration and locomotor activity, using a 2 (treatment) x 3 (strain) x 2 (experimenter) x 2 (balanced/unbalanced pool) factorial design, including all interactions. (DOCX)

S1 Raw data (XLSX)
