# Supplementary material for: Incorporating inter-individual variability in experimental design improves the quality of results of animal experiments
Source: PLoS One. 2021 Aug 5;16(8):e0255521. doi: 10.1371/journal.pone.0255521 (PMC8341614; doi:10.1371/journal.pone.0255521)
Supplement: S1 Table — (DOCX) [file pone.0255521.s001.docx]

**Table S1.** Behavioral variables measured in mHB and used for composition of z-scores in this paper.

| *Motivational system/Behavioral dimension* | *Behavioral variable* | *Directionality*  *z-score^1^* |
| --- | --- | --- |
| *Anxiety related behavior* |  |  |
| - *Avoidance behavior* | Total number of board entries  Latency until first board entry  Percentage of time spent on the board | -z  z  -z |
| *Activity* |  |  |
| - *Exploration* | Total number of rearings in the box  Latency until first rearing in the box  Total number of rearings on the board  Latency until first rearing on the board  Total number of hole explorations  Latency until first hole exploration  Total number of hole visits  Latency until first hole visit | z  -z  z  -z  z  -z  z  -z |
| - *Locomotion* | Total number of line crossings  Latency until first line crossing | z  -z |

^1^ Directionality of z-score: z-scores were adjusted as such that increase of value reflects increase

in corresponding behavioral dimension: [Z]=regular z-score; [-Z]=adjusted z-score.
