# Supplementary material for: Incorporating inter-individual variability in experimental design improves the quality of results of animal experiments
Source: PLoS One. 2021 Aug 5;16(8):e0255521. doi: 10.1371/journal.pone.0255521 (PMC8341614; doi:10.1371/journal.pone.0255521)
Supplement: S2 Table — (DOCX) [file pone.0255521.s002.docx]

**Table S2.** Overview of Dunn-Sidak corrected values for α in *post hoc* comparisons.

| Results section | Analysis type | GLMM effect | Post hoc comparisons/contrasts | γ | Adjusted α |
| --- | --- | --- | --- | --- | --- |
| 2.1. Cluster analyses | LMM | Cluster (A/B) x Trial (T) | A-T1 vs A-T5; B-T1 vs B-T5; A-T1 vs B-T1; A-T5 vs B-T5 | 2 | 0.025321 |
|  | LMM |  | A-T2 vs B-T2; A-T3 vs B-T3; A-T4 vs B-T4 | 1 | 0.05 |
| 2.2.1 | GLM | Strain | C vs B6N; C vs 129S2; B6N vs 129S2 | 2 | 0.025321 |
| 2.2.2 | GLM | Strain | C vs B6N; C vs 129S2; B6N vs 129S2 | 2 | 0.025321 |
|  | GLM | Strain x Treatment (1 = dex/0 = saline) | C-1 vs C-0; B6N-1 vs B6N-0; 129S2-1 vs 129S2-0; C-1 vs B6N-1; C-1 vs 129S2-1; B6N-1 vs 129S2-1; C-0 vs B6N-0; C-0 vs 129S2-0; B6N-0 vs 129S2-0 | 3 | 0.01692 |
