# Supplementary material for: Incorporating inter-individual variability in experimental design improves the quality of results of animal experiments
Source: PLoS One. 2021 Aug 5;16(8):e0255521. doi: 10.1371/journal.pone.0255521 (PMC8341614; doi:10.1371/journal.pone.0255521)
Supplement: S3 Table — Mean integrated behavioral z-score and corresponding 95% confidence interval for each cluster (A/B) on each trial (1–5) for avoidance behavior, exploration and locomotion. (DOCX) [file pone.0255521.s003.docx]

**Table S3.** Mean integrated behavioral z-score and corresponding 95% confidence interval for each cluster (A/B) on each trial (1-5) for avoidance behavior, exploration and locomotion.

|  |  | **Cluster A** | | | **Cluster B** | | |
| --- | --- | --- | --- | --- | --- | --- | --- |
| **Dimension** | *trial* | *mean* | *ci_lower* | *ci_upper* | *mean* | *ci_lower* | *ci_upper* |
| Avoidance behavior | 1 | -0.24308 | -0.41723 | -0.06892 | 0.585057 | 0.418256 | 0.751857 |
|  | 2 | -0.46376 | -0.60747 | -0.32005 | 0.114297 | -0.05352 | 0.282115 |
|  | 3 | 0.035222 | -0.11867 | 0.189111 | -0.13797 | -0.30511 | 0.029171 |
|  | 4 | 0.309293 | 0.163164 | 0.455422 | -0.325 | -0.48178 | -0.16823 |
|  | 5 | 0.483182 | 0.34714 | 0.619224 | -0.40019 | -0.55286 | -0.24751 |
| Exploration | 1 | -0.1861 | -0.25953 | -0.11267 | -0.37601 | -0.4554 | -0.29662 |
|  | 2 | 0.066687 | -0.04843 | 0.1818 | -0.06227 | -0.1757 | 0.051154 |
|  | 3 | -0.02464 | -0.14377 | 0.094501 | 0.215273 | 0.083324 | 0.347222 |
|  | 4 | -0.18049 | -0.30064 | -0.06034 | 0.452915 | 0.302902 | 0.602928 |
|  | 5 | -0.27097 | -0.39648 | -0.14545 | 0.577163 | 0.43668 | 0.717645 |
| Locomotion | 1 | -0.06232 | -0.21351 | 0.088868 | -0.20182 | -0.40712 | 0.003474 |
|  | 2 | -0.09902 | -0.26566 | 0.067618 | 0.275214 | 0.092459 | 0.457969 |
|  | 3 | -0.21974 | -0.39384 | -0.04563 | 0.438686 | 0.329698 | 0.547674 |
|  | 4 | -0.27481 | -0.44041 | -0.10921 | 0.456813 | 0.343232 | 0.570394 |
|  | 5 | -0.38321 | -0.56038 | -0.20605 | 0.439368 | 0.302839 | 0.575897 |
