# Supplementary material for: Incorporating inter-individual variability in experimental design improves the quality of results of animal experiments
Source: PLoS One. 2021 Aug 5;16(8):e0255521. doi: 10.1371/journal.pone.0255521 (PMC8341614; doi:10.1371/journal.pone.0255521)
Supplement: S4 Table — Post hoc tests comparing either (I) the estimated marginal means between trials 1 and 5 (adjusted α = 0.016952) for avoidance behavior, exploration and locomotion and (II) cluster differences on each trial for avoidance behavior, exploration and locomotion (adjusted α = 0.016952). Significant comparisons are highlighted in bold. (DOCX) [file pone.0255521.s004.docx]

**Table S4**. *Post hoc* tests comparing either (I) the estimated marginal means between trials 1 and 5 (adjusted α = 0.016952) for avoidance behavior, exploration and locomotion and (II) cluster differences on each trial for avoidance behavior, exploration and locomotion (adjusted α = 0.016952). Significant comparisons are highlighted in bold.

| **(I) Dimension** |  | **Estimate ± SEM** | ***t_(df)_*** | ***P*** | **Cohens *d* [95% CI]** |
| --- | --- | --- | --- | --- | --- |
| Avoidance |  |  |  |  |  |
| Trial 1 vs 5 |  |  |  |  |  |
|  | A | -0.726 ± 0.96 | -7.593_(708)_ | **< 0.0001** | -1.015 [-1.283, -0.747] |
|  | B | 0.985 ± 0.96 | 10.288_(708)_ | **< 0.0001** | 1.377 [1.105, 1.650] |
| Exploration |  |  |  |  |  |
| Trial 1 vs 5 |  |  |  |  |  |
|  | A | 0.085 ± 0.05 | 1.583_(708)_ | 0.1138 | 0.319 [-0.077, 0.716] |
|  | B | -0.953 ± 0.06 | -15.274_(708)_ | **< 0.0001** | -3.588 [-4.085, -3.090] |
| Locomotion |  |  |  |  |  |
| (rank transformed) |  |  |  |  |  |
| Trial 1 vs 5 |  |  |  |  |  |
|  | A | 98.06 ± 23.1 | 4.250_(708)_ | **< 0.0001** | 0.498 [0.267, 0.730] |
|  | B | -252.68 ± 32.6 | -7.740_(708)_ | **< 0.0001** | -1.285 [-1.618, -0.952] |
| **(II) Dimension** |  | **Estimate ± SEM** | ***t_(df)_*** | ***P*** | **Cohen’s *d* [95% CI]** |
| Avoidance |  |  |  |  |  |
| A vs B | Trial 1 | -0.828 ± 0.111 | -7.430_(177)_ | **< 0.0001** | -1.158 [-1.488, -0.827] |
|  | Trial 2 | -0.578 ± 0.111 | -5.186_(177)_ | **< 0.0001** | -0.808 [-1.127, -0.489] |
|  | Trial 3 | 0.173 ± 0.111 | 1.554_(177)_ | 0.1220 | 0.242 [-0.066, 0.551] |
|  | Trial 4 | 0.634 ± 0.111 | 5.691_(177)_ | **< 0.0001** | 0.887 [0.565, 1.208] |
|  | Trial 5 | 0.883 ± 0.111 | 7.926_(177)_ | **< 0.0001** | 1.235 [0.901, 1.569] |
| Exploration |  |  |  |  |  |
| A vs B | Trial 1 | 0.190 ± 0.057 | 3.318_(177)_ | **0.0011**** | 0.715 [0.283, 1.147] |
|  | Trial 2 | 0.129 ± 0.078 | 1.658_(177)_ | 0.0992 | 0.485 [-0.095, 1.066] |
|  | Trial 3 | -0.240 ± 0.083 | -2.900_(177)_ | **0.0042**** | -0.903 [-1.525, -0.281] |
|  | Trial 4 | -0.633 ± 0.095 | -6.658_(177)_ | **< 0.0001** | -2.384 [-3.134, -1.635] |
|  | Trial 5 | -0.848 ± 0.100 | -8.452_(177)_ | **< 0.0001** | -3.192 [-4.009, -2.375] |
| Locomotion  (rank transformed) |  |  |  |  |  |
| A vs B | Trial 1 | 43.7 ± 46.0 | 0.949_(177)_ | 0.3438 | 0.222 [-0.240, 0.685] |
|  | Trial 2 | -152.1 ± 35.7 | -4.260_(177)_ | **< 0.0001** | -0.774 [-1.140, -0.406] |
|  | Trial 3 | -227.4 ± 33.6 | -6.774_(177)_ | **< 0.0001** | -1.157 [-1.510, -0.798] |
|  | Trial 4 | -272.1 ± 31.8 | -8.551_(177)_ | **< 0.0001** | -1.384 [-1.730, -1.033] |
|  | Trial 5 | -307.1 ± 31.2 | -9.851_(177)_ | **< 0.0001** | -1.562 [-1.910, -1.208] |
