# Supplementary material for: Incorporating inter-individual variability in experimental design improves the quality of results of animal experiments
Source: PLoS One. 2021 Aug 5;16(8):e0255521. doi: 10.1371/journal.pone.0255521 (PMC8341614; doi:10.1371/journal.pone.0255521)
Supplement: S5 Table — (DOCX) [file pone.0255521.s005.docx]

**Table S5**. Raw integrated z-scores (mean ± 95% confidence interval) of groups (n=8/group) that were compared in GLMM’s to test the effects of treatment, strain, pool and experimenter on avoidance behavior, exploration and locomotor activity, using a 2 (treatment) x 3 (strain) x 2 (experimenter) x 2 (balanced/unbalanced pool) factorial design, including all interactions.

| **Dimension** | **Main effect/condition** | **n** | **mean** | **ci_lower** | **ci_upper** |
| --- | --- | --- | --- | --- | --- |
| Avoidance behavior | treatment - saline | 48 | -0.16072 | -0.3636 | -0.52433 |
|  | treatment - dex | 48 | 0.160721 | -0.11315 | 0.047571 |
|  | strain - 129S2 | 32 | -0.2489 | -0.5847 | -0.8336 |
|  | strain - C | 32 | -0.02607 | -0.33485 | -0.36093 |
|  | strain - B6N | 32 | 0.274979 | 0.039869 | 0.510089 |
|  | pool - unbalanced | 48 | 0.188684 | -0.06204 | 0.126641 |
|  | pool - balanced | 48 | -0.18868 | -0.41588 | -0.60457 |
|  | experimenter - I | 48 | 0.228397 | -0.0024 | 0.225996 |
|  | experimenter - II | 48 | -0.2284 | -0.46998 | -0.69838 |
| Exploration | treatment - saline | 48 | 0.145162 | -0.0353 | 0.109859 |
|  | treatment - dex | 48 | -0.14516 | -0.29899 | -0.44415 |
|  | strain - 129S2 | 32 | -0.29773 | -0.39063 | -0.68837 |
|  | strain - C | 32 | -0.10129 | -0.28281 | -0.3841 |
|  | strain - B6N | 32 | 0.39902 | 0.146633 | 0.545652 |
|  | pool - unbalanced | 48 | -0.10192 | -0.25331 | -0.35523 |
|  | pool - balanced | 48 | 0.101924 | -0.08557 | 0.016359 |
|  | experimenter - I | 48 | -0.15937 | -0.30941 | -0.46877 |
|  | experimenter - II | 48 | 0.159367 | -0.02222 | 0.137148 |
| Locomotion | treatment - saline | 48 | 0.216326 | 0.032184 | 0.24851 |
|  | treatment - dex | 48 | -0.21633 | -0.48164 | -0.69796 |
|  | strain - 129S2 | 32 | -0.46171 | -0.76736 | -1.22907 |
|  | strain - C | 32 | -0.057 | -0.27732 | -0.33432 |
|  | strain - B6N | 32 | 0.518705 | 0.283088 | 0.801793 |
|  | pool - unbalanced | 48 | 0.021259 | -0.2095 | -0.18825 |
|  | pool - balanced | 48 | -0.02126 | -0.26421 | -0.28547 |
|  | experimenter - I | 48 | -0.13789 | -0.42482 | -0.5627 |
|  | experimenter - II | 48 | 0.137889 | -0.02568 | 0.112207 |
