# Supplementary material for: Incorporating inter-individual variability in experimental design improves the quality of results of animal experiments
Source: PLoS One. 2021 Aug 5;16(8):e0255521. doi: 10.1371/journal.pone.0255521 (PMC8341614; doi:10.1371/journal.pone.0255521)
Supplement: S6 Table — Post hoc tests comparing (a) the estimated marginal means between strains (adjusted α = 0.025321) for each behavioral dimension, on the total dataset, so balanced and unbalanced combined, section 2.2.1 (b) strain differences on each behavioral dimension(adjusted α = 0.025321) for the balanced data only and (c) strain differences on avoidance behavior and locomotion (adjusted α = 0.025321) or strain comparisons within treatment/within strain comparisons between treatments (adjusted α = 0.016952) for exploration. Significant comparisons are highlighted in bold. (DOCX) [file pone.0255521.s006.docx]

**Table S6**. *Post hoc* tests comparing (a) the estimated marginal means between strains (adjusted α = 0.025321) for each behavioral dimension, on the total dataset, so balanced and unbalanced combined, section 2.2.1 (b) strain differences on each behavioral dimension(adjusted α = 0.025321) for the balanced data only and (c) strain differences on avoidance behavior and locomotion (adjusted α = 0.025321) or strain comparisons within treatment/within strain comparisons between treatments (adjusted α = 0.016952) for exploration. Significant comparisons are highlighted in bold.

| **(a) Balanced and unbalanced pool combined: post hoc comparisons** | | | | | |
| --- | --- | --- | --- | --- | --- |
| Dimension |  | Estimate ± SEM | z | P | Cohens d [95% CI] |
| Avoidance |  |  |  |  |  |
| Strain effect | 129S2 vs C | -0.192 ± 0.205 | -0.940 | 0.3471 | -0.239 [-0.738, 0.260] |
|  | 129S2 vs B6N | -0.465 ± 0.213 | -2.184 | 0.0290 | -0.577 [-1.104, -0.050] |
|  | C vs B6N | -0.273 ± 0.204 | -1.335 | 0.1819 | -0.338 [-0.838, 0.162] |
| Exploration |  |  |  |  |  |
| Strain effect | 129S2 vs C | -0.220 ± 0.140 | -1.571 | 0.1161 | -0.399 [-0.901, 0.103] |
|  | 129S2 vs B6N | -0.730 ± 0.145 | -5.024 | **< 0.0001** | -1.327 [-1.891, -0.763] |
|  | C vs B6N | -0.511 ± 0.139 | -3.663 | **0.0002** | -0.928 [-1.499, -0.408] |
| Locomotion  (rank transformed) |  |  |  |  |  |
| Strain effect | 129S2 vs C | -15.8 ± 5.28 | -2.994 | **0.0028** | -0.760 [-1.270, -0.246] |
|  | 129S2 vs B6N | -41.6 ± 5.50 | -7.563 | **< 0.0001** | -2.000 [-2.620, -1.381] |
|  | C vs B6N | -25.8 ± 5.27 | -4.886 | **< 0.0001** | -1.240 [-1.780, -0.700] |
|  |  |  |  |  |  |
| **(b) Balanced pool only: post hoc comparisons** | | | | | |
| Dimension |  | Estimate ± SEM | z | P | Cohens d [95% CI] |
| Avoidance |  |  |  |  |  |
| Strain effect | 129S2 vs C | -0.235 ± 0.316 | -0.744 | 0.4568 | -0.317 [-1.155, 0.521] |
|  | 129S2 vs B6N | -0.377 ± 0.333 | -1.130 | 0.2587 | -0.507 [-1.396, 0.382] |
|  | C vs B6N | -0.141 ± 0.264 | -0.535 | 0.5927 | -0.190 [-0.889, 0.509] |
| Exploration |  |  |  |  |  |
| Strain effect | 129S2 vs C | -0.352 ± 0.225 | -1.563 | 0.1181 | -0.655 [-1.520, 0.185] |
|  | 129S2 vs B6N | -0.909 ± 0.237 | -3.828 | **0.0001** | -1.719 [-2.690, -0.743] |
|  | C vs B6N | -0.557 ± 0.188 | -2.961 | **0.0031** | -1.053 [-1.800, -0.310] |
| Locomotion  (rank transformed) |  |  |  |  |  |
| Strain effect | 129S2 vs C | -9.86 ± 4.51 | -2.183 | 0.0290 | -0.93 [-1.79, -0.06] |
|  | 129S2 vs B6N | -23.83 ± 4.76 | -5.006 | **< 0.0001** | -2.25 [-3.29, -1.209] |
|  | C vs B6N | -13.98 ± 3.77 | -3.702 | **0.0002** | -1.32 [-2.09, -0.550] |
| **(c) Unbalanced pool only: post hoc comparisons** | | | | |  |
| Dimension |  | Estimate ± SEM | z | P | Cohens d [95% CI] |
| Avoidance |  |  |  |  |  |
| Strain effect |  |  |  |  |  |
|  | 129S2 vs C | -0.088 ± 0.331 | -0.267 | 0.7893 | -0.096 [-0.805, 0.612] |
|  | 129S2 vs B6N | -0.495 ± 0.324 | -1.528 | 0.1266 | -0.540 [-1.246, 0.165] |
|  | C vs B6N | -0.407 ± 0.333 | -1.223 | 0.2215 | -0.444 [-1.163, 0.276] |
| Exploration |  |  |  |  |  |
| Strain x Treatment interaction |  |  |  |  |  |
| Treatment (T) | 129S2 vs C | -0.012 ± 0.219 | 0.056 | 0.9556 | -0.028 [-0.961, 1.018] |
|  | 129S2 vs B6N | -0.153 ± 0.217 | -0.708 | 0.4793 | -0.354 [-1.338, 0.630] |
|  | C vs B6N | -0.166 ± 0.220 | -0.753 | 0.4514 | -0.382 [-1.381, 0.617] |
| Control (C) | 129S2 vs C | -0.273 ± 0.219 | -1.245 | 0.2133 | -0.630 [-1.634, 0.374] |
|  | 129S2 vs B6N | -1.097 ± 0.217 | -5.062 | **< 0.0001** | -2.531 [-3.691, -1.371] |
|  | C vs B6N | -0.824 ± 0.219 | -3.757 | **0.0002** | -1.901 [-2.997, -0.805] |
| C versus T | 129S2 | -0.031 ± 0.217 | -0.144 | 0.8852 | -0.072 [-1.052, 0.908] |
|  | C | 0.254 ± 0.217 | 1.171 | 0.2415 | 0.586 [-0.405, 1.576] |
|  | B6N | 0.913 ± 0.217 | 4.207 | **< 0.0001** | 2.105 [0.997, 3.213] |
| Locomotion  (rank transformed) |  |  |  |  |  |
| Strain effect | 129S2 vs C | -6.36 ± 3.85 | -1.653 | 0.0983 | -0.597 [-1.32, 0.126] |
|  | 129S2 vs B6N | -18.26 ± 3.77 | -4.845 | **< 0.0001** | -1.713 [-2.52, -0.903] |
|  | C vs B6N | -11.89 ± 3.87 | -3.076 | **0.0021** | -1.116 [-1.88, -0.354] |
