# Supplementary material for: Incorporating inter-individual variability in experimental design improves the quality of results of animal experiments
Source: PLoS One. 2021 Aug 5;16(8):e0255521. doi: 10.1371/journal.pone.0255521 (PMC8341614; doi:10.1371/journal.pone.0255521)
Supplement: S7 Table — (DOCX) [file pone.0255521.s007.docx]

**Table S7**. Raw integrated z-scores (mean ± 95% confidence interval) of groups (*n* = 4/group) that were compared in GLMM’s to test the effects of treatment, strain, pool and experimenter on avoidance behavior, exploration and locomotor activity, using a 2 (treatment) x 3 (strain) x 2 (experimenter) x 2 (balanced/unbalanced pool) factorial design, including all interactions.

|  |  |  | Design: balanced | | | Design: *not* balanced | | |
| --- | --- | --- | --- | --- | --- | --- | --- | --- |
| Dimension | Experimenter | Strain/treatment | mean | ci_lower | ci_upper | mean | ci_lower | ci_upper |
| Avoidance behavior | Exp I | 129S2 -saline | 0.023019 | -1.61607 | 1.662108 | 0.200246 | -1.38651 | 1.787001 |
|  |  | 129S2-dex | 0.129693 | -1.43332 | 1.692703 | 0.016335 | -1.94767 | 1.980338 |
|  |  | C-saline | 0.101698 | -1.47017 | 1.673563 | -0.03734 | -1.26342 | 1.188748 |
|  |  | C-dex | 0.441862 | -0.82784 | 1.711569 | 0.193076 | -1.60137 | 1.987524 |
|  |  | B6N-saline | 0.429354 | -0.13032 | 0.989028 | 0.09654 | -0.59096 | 0.784039 |
|  |  | B6N-dex | 0.625128 | -0.28483 | 1.535088 | 0.571633 | -0.73361 | 1.876876 |
|  | Exp II | 129S2 -saline | -0.69476 | -1.12455 | -0.26498 | -0.37338 | -1.97587 | 1.229113 |
|  |  | 129S2-dex | -0.73457 | -1.73569 | 0.266541 | -0.59157 | -2.28593 | 1.102792 |
|  |  | C-saline | -0.55749 | -1.06447 | -0.05052 | -0.5685 | -1.24379 | 0.10679 |
|  |  | C-dex | 0.316467 | -1.34841 | 1.981347 | -0.08268 | -2.19269 | 2.027338 |
|  |  | B6N-saline | -0.27818 | -0.78015 | 0.223796 | -0.24184 | -0.77131 | 0.287622 |
|  |  | B6N-dex | 0.197786 | -1.37734 | 1.772915 | 0.817472 | 0.487122 | 1.147822 |
| Exploration | Exp I | 129S2 -saline | -0.39387 | -0.87417 | 0.086425 | -0.4229 | -0.89838 | 0.052583 |
|  |  | 129S2-dex | -0.50552 | -0.85873 | -0.1523 | -0.43842 | -0.92738 | 0.050539 |
|  |  | C-saline | -0.25477 | -0.88672 | 0.377188 | -0.222 | -0.76146 | 0.317457 |
|  |  | C-dex | -0.33452 | -0.79876 | 0.129717 | -0.32847 | -0.7302 | 0.07327 |
|  |  | B6N-saline | 0.187644 | -0.33855 | 0.713839 | 0.767557 | -0.60851 | 2.143629 |
|  |  | B6N-dex | 0.242245 | -0.85694 | 1.341427 | -0.23107 | -0.678 | 0.21587 |
|  | Exp II | 129S2 -saline | -0.16467 | -0.31148 | -0.01785 | -0.13366 | -0.59896 | 0.33163 |
|  |  | 129S2-dex | -0.24404 | -0.48126 | -0.00681 | -0.05553 | -0.21117 | 0.100119 |
|  |  | C-saline | 0.352888 | -0.74396 | 1.449732 | 0.287712 | -0.26056 | 0.835983 |
|  |  | C-dex | -0.1803 | -1.0436 | 0.682995 | -0.12176 | -1.29527 | 1.051738 |
|  |  | B6N-saline | 0.8984 | -0.08546 | 1.882258 | 0.870524 | 0.261631 | 1.479417 |
|  |  | B6N-dex | 0.396512 | -1.26627 | 2.05929 | 0.028014 | -0.48706 | 0.543084 |
| Locomotion | Exp I | 129S2 -saline | -0.30246 | -0.84295 | 0.238036 | -0.03936 | -0.55794 | 0.479215 |
|  |  | 129S2-dex | -1.14415 | -3.43442 | 1.146118 | -1.3907 | -3.63961 | 0.858205 |
|  |  | C-saline | -0.14697 | -1.39312 | 1.099179 | 0.038583 | -0.8603 | 0.937462 |
|  |  | C-dex | -0.21732 | -2.33342 | 1.898771 | 0.025043 | -0.884 | 0.934084 |
|  |  | B6N-saline | 0.851491 | 0.214075 | 1.488907 | 0.57257 | 0.083174 | 1.061966 |
|  |  | B6N-dex | 0.473804 | -0.11642 | 1.064024 | -0.39373 | -2.17827 | 1.390809 |
|  | Exp II | 129S2 -saline | -0.35352 | -1.28484 | 0.57779 | -0.11184 | -0.58074 | 0.357052 |
|  |  | 129S2-dex | -0.25024 | -0.53849 | 0.038012 | -0.06912 | -0.55721 | 0.418971 |
|  |  | C-saline | -0.01579 | -0.37472 | 0.343136 | 0.194522 | -0.21018 | 0.599226 |
|  |  | C-dex | 0.012291 | -0.74927 | 0.773856 | -0.34784 | -0.83812 | 0.142431 |
|  |  | B6N-saline | 0.875258 | 0.393329 | 1.357187 | 1.045723 | -0.19156 | 2.283011 |
|  |  | B6N-dex | 0.217619 | -0.45245 | 0.887684 | 0.476164 | 0.132941 | 0.819387 |
